# HPN-07, a free radical spin trapping agent, protects against functional, cellular and electrophysiological changes in the cochlea induced by acute acoustic trauma

**DOI:** 10.1371/journal.pone.0183089

**Published:** 2017-08-23

**Authors:** Donald Ewert, Ning Hu, Xiaoping Du, Wei Li, Matthew B. West, Chul-Hee Choi, Robert Floyd, Richard D. Kopke

**Affiliations:** 1 Hough Ear Institute, Oklahoma City, OK, United States of America; 2 Department of Biology, University of Iowa, Iowa City, IA, United States of America; 3 Audiology & Speech-Language Pathology & Research Institute of Biomimetic Sensory Control, College of Medical Sciences, Catholic University of Daegu, Gyeongsangbuk-do, South Korea; 4 Oklahoma Medical Research Foundation, Oklahoma City, OK, United States of America; 5 Departments of Physiology and Otolaryngology, University of Oklahoma Health Sciences Center, Oklahoma City, OK, United States of America; Kyoto Daigaku, JAPAN

## Abstract

Oxidative stress is considered a major cause of the structural and functional changes associated with auditory pathologies induced by exposure to acute acoustic trauma AAT). In the present study, we examined the otoprotective effects of 2,4-disulfophenyl-*N*-tert-butylnitrone (HPN-07), a nitrone-based free radical trap, on the physiological and cellular changes in the auditory system of chinchilla following a six-hour exposure to 4 kHz octave band noise at 105 dB SPL. HPN-07 has been shown to suppress oxidative stress in biological models of a variety of disorders. Our results show that administration of HPN-07 beginning four hours after acoustic trauma accelerated and enhanced auditory/cochlear functional recovery, as measured by auditory brainstem responses (ABR), distortion product otoacoustic emissions (DPOAE), compound action potentials (CAP), and cochlear microphonics (CM). The normally tight correlation between the endocochlear potential (EP) and evoked potentials of CAP and CM were persistently disrupted after noise trauma in untreated animals but returned to homeostatic conditions in HPN-07 treated animals. Histological analyses revealed several therapeutic advantages associated with HPN-07 treatment following AAT, including reductions in inner and outer hair cell loss; reductions in AAT-induced loss of calretinin-positive afferent nerve fibers in the spiral lamina; and reductions in fibrocyte loss within the spiral ligament. These findings support the conclusion that early intervention with HPN-07 following an AAT efficiently blocks the propagative ototoxic effects of oxidative stress, thereby preserving the homeostatic and functional integrity of the cochlea.

## Introduction

Hearing loss due to occupational noise exposure is a common form of sensorineural hearing loss (SNHL) in industrialized countries. The Centers for Disease Control and Prevention estimates that ten million people in the U.S. have a noise-related hearing loss, and twenty-two million workers are exposed to potentially damaging noise each year [1]. Acute acoustic traumas (AAT) induce the accumulation of reactive oxygen species (ROS), reactive nitrogen species (RNS), and other free radical species [2], which are associated with metabolic damage to the cellular components of the inner ear [3,4,5,6]. ROS directly damage cell structures by reacting with proteins, lipids, and DNA and act as signaling molecules that up-regulate pro-apoptotic gene expression. Accordingly, much emphasis has been placed on discovering antioxidant compounds that can neutralize highly reactive oxygen species to prevent and/or treat damage to the cochlea [6,7,8,9,10,11].

In this study, we examine the otoprotective effects of disodium 2,4-disulfophenyl-N-tert-butylnitrone (HPN-07), a nitrone-based spin trapping agent of free radical species (previously called NXY-059, a disulfonyl-phenyl derivative of phenyl-N-tert-butylnitrone, or PBN) [7,12]. HPN-07 is used to trap free radicals in chemical and biochemical system and has been shown to be neuroprotective in several preclinical models of ischemic stroke [13] and in traumatic brain injury [14]. Moreover, it has been demonstrated that HPN-07 is safe and well-tolerated in phase III clinical trials conducted for the treatment of stroke [15, 16, 17, 18].

Our previous studies demonstrated that a combination of HPN-07 and *N*-acetylcysteine (NAC) is capable of reducing both temporary and permanent auditory brainstem response (ABR) and distortion product otoacoustic emission (DPOAE) threshold shifts and hair cell loss in rats after exposure to blast trauma [19] or intense noise [20]. Several studies have previously explored the otoprotective effects of NAC alone in both animals [10,21,22,23] and humans [24, 25, 26]. The present study is the first evaluation of the effects of HPN-07 as a single therapeutic agent for protecting against acute and progressive cochlear damage induced by AAT.

In this study, in addition to using ABR and DPOAEs to evaluate auditory function, we have extended our analyses to include measurements of three potentials that are generated in the cochlea: the endocochlear potential (EP), cochlear microphonic (CM), and compound action potential (CAP) [27]. The ABR consists of the far-field potentials from the auditory nervous system that are generated by the combined neural activity in the ear, auditory nerve, and auditory nuclei and fiber tracts of the ascending auditory pathways. DPOAEs are evoked otoacoustic emissions generated by the outer hair cells (OHC) in response to exposure to pure tones. The EP corresponds to the resting potential of the *scala media* of the cochlea originating from the *stria vascularis* (SV). The EP reflects the homeostasis of the inner ear environment, and plays an essential role in the transduction of sound waves into nerve impulses by the hair cells. CM is the near-field potentials generated as receptor currents flow through the outer hair cells. The CAP results from the synchronous firing of spiral ganglion neurons in response to sound stimulation and provides information about the integrity of the auditory nerve. Previous studies have found noise-induced changes in cochlear potentials to be noise intensity-dependent, with spontaneous recovery of cochlear potentials often occurring within 5–20 days following an AAT [28,29,30]. Long term studies on the recovery of these parameters beyond 28d following noise exposure have not been reported. EP reduction has been shown to be a factor in the acute [31] and permanent phase [32] of hearing loss. In the latter study in chinchilla suppression of EP up to 28 d after noise exposure was associated with disruption of the reticular lamina. One of the aims of this study is to determine if HPN-07 can affect the noise induced changes in the cochlear architecture that are associated with the changes in the endocochlear potentials.

The overall aims of this study are to gain new insights into mechanisms that contribute to the functional and electrophysiological changes in the cochlea following AAT and to evaluate therapeutic impact of HPN-07 intervention. To this end, we demonstrate quantifiable changes in hair cell counts and biomarkers for neurons in the organ of Corti and spiral ganglia that can be correlated with the changes in electrophysiology.

## Methods and materials

### Experimental subjects

Three-year-old healthy female chinchillas weighing 550–750 g (Moulton Chinchilla Ranch, Rochester, MN; and Ryerson Chinchilla Ranch, Plymouth, OH) were used in this project. Animals were housed in plastic cages in the animal facility at the Oklahoma Medical Research Foundation (OMRF). They were allowed free access to a standard chinchilla diet (Mazuri Chinchilla Diet, 5MO1, PM1 Nutrition International Inc., Brentwood, MO) and tap water throughout the time course of experimentation. The experimental protocol was reviewed and approved by the Institutional Animal Care and Use Committees of the Office of Naval Research and OMRF in accordance with the Animal Welfare Act and the Guide for the Care and Use of Laboratory Animals, prepared by the Institute of Laboratory Animal Resources, National Academy of Sciences–National Research Council.

### Experimental groups

A total of 72 chinchillas were divided into three experimental groups: a) normal (naïve, control group without noise exposure); b) control (noise-exposed without treatment); c) treated (noise-exposed treated with HPN-07). Four separate time-limited cohorts containing animals assigned to each of three groups (six animals per group) were followed in separate studies. Each study of 18 animals was terminated at either at 3, 10, 21 or 180 days after noise exposure. The latter group in which the same 18 animals were followed for 180 days is referred to as the longitudinal cohort. Non-invasive analyses (i.e. ABR and DPOAE) were performed at all time points, and the remaining tests (i.e. EP, CM, and CAP) were conducted at the terminal time point in each cohort. Following terminal testing, the sedated animals were perfused with fixative, and the cochleae extracted for quantification of hair cells and biomarker studies as described below. The actual numbers of animals or specimens examined are presented in the "Results" section and figures.

### Noise exposure

Animals were exposed to a 105 dB SPL octave-band noise centered at 4 kHz for six hours in a sound isolation booth (Industrial Acoustics Company, New York, NY). The noise was produced using a compression driver (model 2247H JBL, Northridge, CA), connected to a square-cross shaped horn (Model 2354, JBL) that was suspended from the ceiling of the sound booth and positioned directly above the animals at a distance of 100 cm. The signal for noise production was digitally generated and filtered by an enhanced real-time processor (model RP2.1, RPvds Ex version 5.4, Tucker Davis Technologies, Alachua, FL) and amplified with a power amplifier (model PLX 3402, QSC Power Amplifier, Costa Mesa, CA). Noise levels were calibrated and measured with a condenser microphone (microphone unit type 4133, Bruel &Kjaer, Norcross, GA), which was operated using the PULSE software system (type 2804, PULSE LabShop Version 10, Bruel & Kjaer, Norcross, GA), including a Fast Fourier Transform (FFT) analyzer and a constant percentage bandwidth (CPB) analyzer.

For each noise exposure, two animals were restrained separately with a breeding collar in a plastic cage and positioned head-to-head around the center axis of the speaker. The noise level was continously monitored with the above-mentioned condenser microphone placed at the center of the space between the two animals at the approximate level of the animals' ears. The variations of noise levels between the centrally located microphone and the animals’ ears and across time were less than 1dB.

### Drug administration

HPN-07, provided at greater than 98.5% purity by APAC Pharmaceuticals LLC 222 (Columbia, MD), was dissolved in physiological saline and administrated by intraperitoneal injection beginning four hours after noise exposure and subsequently twice daily for the next two days. The experimental dosage of HPN-07 was 300 mg/kg per administration.

### Hearing assessment schedule

Repeated non-invasive assessments of auditory function were conducted before and during the course of the experiment by measuring the ABR and DPOAE. Baseline ABR and DPOAE levels were obtained for each animal before noise exposure, followed by measurements at 3d, 10d, 21d, and 180d after noise exposure. Three tests of the auditory systems were performed as terminal procedures: CAP, CM, and EP. All measurements were conducted in a sound isolation booth (Industrial Acoustics Company, New York, NY) under ketamine (20 mg/kg) and xylazine (2.0 mg/kg) anesthesia. Supplemental doses (1/3 of initial dose) of anesthesia were given every 45 min or based on a toe pinch reflex. The body temperature was maintained at the core temperature of 37^0^ C using an electric blanket (DC temperature controller, FHC). For terminal procedures, a tracheotomy was performed, and a ventilation tube was inserted into the trachea to allow for spontaneous breathing. The round window (RW) niche and the bony cochlear turns were exposed through a ventral approach.

### Auditory brainstem response (ABR)

ABR testing was preformed as previously described (33). The recording electrode configuration was an active needle electrode placed at the midline of the vertex of the skull along with a reference electrode and a ground electrode at the ipsilateral and contralateral mastoid areas of the stimulus ear, respectively. A computer-aided system [SmartEP window USB version 3.98, Intelligent Hearing Systems (IHS), Miami, FL] was used to generate auditory stimuli and display and record electrical responses. Acoustic stimuli were tone pips (5 ms duration with Blackman rise and fall ramp) at frequencies of 0.5, 1, 2, 4, 6, and 8 kHz, as well as click stimuli. All acoustic stimuli were delivered to the external auditory meatus through an insert earphone (IHS) 3A insert earphone. The electrical responses obtained from the electrodes were amplified (×100,000), filtered (100–3000 Hz), averaged (128–256 sweeps), and digitized through an A/D converter on a signal processing board. Stimuli were presented at 19.3/s stimulus rate and alternative polarities and in 10 dB descending steps until near the threshold level followed by 5 dB steps to determine the threshold. ABR threshold was defined as the lowest stimulus level that evoked a repeatable waveform based on an identifiable ABR wave III.

### Distortion product otoacoustic emission (DPOAE)

DPOAE tests were performed as previously described (34). DPOAEs for both ears of each animal were measured using the Smart DPOAE system (IHS) under the same anesthesia condition as used for the ABR recordings. Levels of two primary tones to elicit a DPOAE were equal (L1 = L2), and the frequency ratio of the two primary tones (F2/F1) was 1.22. The DPOAE of 2F1-F2 was obtained at two frequency points per octave from 1.0 to 8.0 kHz. There were a total of seven frequency points at each stimulus level (1.1, 1.6, 2.2, 3.1, 4.4, 6.2, and 8.8 kHz at F2 frequency). Stimulus levels ranged from 75 to 10 dB SPL below the threshold level in 5 dB steps. Sixteen sweeps per frequency and eight sweeps per block were set in the Smart DPOAE system. The level of the DPOAE and the related noise floor (average of several spectral sample points around 2F_1_-F_2_) were automatically displayed by the test system, and the DPOAE amplitudes were recorded as both absolute value and signal-to-noise ratio. The noise floor was generally lower than 10dB SPL.

DPOAE threshold was also obtained as an outcome parameter for group comparisons. We used two criterion to define the DPOAE. According to the growth function of DPOAE at each frequency, the threshold of DPOAE was defined as a level producing a DPOAE with both absolute amplitude larger than -10 dB SPL and relative amplitude larger than 6 dB above the noise floor. Similar to ABR threshold shift measurements, DPOAE amplitude shift and threshold shift relative to its own baseline level were also obtained as another outcome parameter.

### Compound action potential (CAP)

The recording electrode configuration for CAP was similar to that used for ABR, except that the reference (inverting) electrode was a silver ball electrode positioned in the RW membrane niche. The recording instrument, parameters and methods were the same as that used for ABR. The assesment of CAP threshold was based on an identiable peak N1. For the CAP threshold change computation, the average level obtained for the normal control animal group was used as the reference.

### Cochlear microphonic (CM)

The recording electrode configuration and recording instrument were the same as that for CAP. Acoustic stimuli were 10 ms duration tone pips with Blackman rise and fall ramps at frequencies of 0.5, 1, 2, 4, 6, 8 kHz. CM waveforms evoked by the rarefaction polarity stimuli were recorded and analyzed. The band pass filter was set from 0.1 to 5 kHz (the highest low-pass cut-off frequency of the test system). The amplitude of CM at each stimulus frequency was obtained using FFT spectrum analysis provided by the SmartEP program (window USB version 3.98) (IHS). The treshold of CM was defined as the lowest stimulus level that evolked a CM amplitude that was twice that of the mean noise floor. The CM level of normal control animals was used a a reference to compute threshold change.

### Endocochlear potential (EP)

After CAP and CM measures, the opening of the bulla was enlarged, and a small hole (about 0.1 mm) was made on the bony wall of the basal cochlear turn under a microscope. A fine needle was then used to expose the spiral ligament. A glass micropipette electrode was produced with a laser micropipette puller Model P-2000 (Sutter Instrument Co. Novato, CA) from a borosilicate class capillary with filament (1.2 mm O.D., 0.94mm I.D., and 10 cm length, item #: BF 120-94-10, Sutter instrument Co. Novato, CA). A micropipette electrode was then filled with 150 mM potassium chloride solution. Impedance of the microelectrode was 20–40 MΩ measured with a microelectrode high impedance meter (Ωmega-Tip Z, World Precision Instrument Inc. Sarasota, FL). The glass micropipette was then connected to a pre-amplifier (HS-9A headstage x0.1, Axon CNS Molecular Devices Corp, Union City, CA), which was mounted on a manual 3D micromanipulator via a 2 HL-U electrode holder (Axon Molecular Devices Corp, Union City, CA) containing an Ag/AgCl wire. The output of the pre-amplifier was connected to a computer-controlled microelectrode amplifier (Axoclamp 900A, Axon Molecular Devices Corp, Union City, CA). A low-pass filter was set at 10 Hz. No capacitance compensation was employed. An Ag/AgCl wire reference was placed in the muscles of the neck. The output of Axoclamp 900 was fed into a computer-controlled soft oscilloscope (PicoScope 2204, Pico Technology, Cambridgeshire, United Kingdom). The signal was displayed at a 1/s sampling rate and recorded/archived for every 500-second period. The DC voltage was set to zero at the surface of the spiral ligament. After a baseline was recorded over the course of approximately 50s, the micropipette electrode was advanced through the spiral ligament into the scala media until a stable EP recording was obtained. After the EP was continuously recorded for about 250 to 300s, the microelectrode was withdrawn from the scala media to the surface of the spiral ligament until the end of the 500s recording period to ensure that the DC drift was less than ± 1mV.

### Histological evaluations

Chinchillas were transcardially perfused with saline followed by 4% paraformaldehyde under deep anesthesia following the final recording session. The temporal bones were removed and fixed overnight with 4% paraformaldehyde, then stored in 0.1M PBS buffer prior to hair cell counting or immunohistochemical analyses. Cochlear micro-dissection was accomplished under a light dissecting microscope. Investigators that performed quantitative analyses among histological preparations were blinded as to the identity of the animal groups.

Cochlear hair cells were counted using surface preparations as previously described [33,34,35]. Cochleae (organs of Corti) designated for hair cell counting were incubated in PBS containing 5 μg/mL of phalloidin conjugated to tetramethylrhodamine isothiocyanate (TRITC) (Sigma, St. Louis, MO) at room temperature for 30 min in the dark. Each OC specimen was then flat mounted in an anti-fade medium on glass slides and was examined under a fluorescence microscope (Olympus BX51, 198 Melville, NY) with 40 **×** magnification. The number of missing hair cells were quantified from the apex to the base in 500 μm segments and plotted as cytocochleograms with the corresponding frequencies represented along the basilar membrane as described in previous reports [21, 36, 37,38]. Hair cells were counted as missing if stereocilia and cuticular plates were absent [39].

For immunohistochemical staining, the cochleae tissues that were not subjected to invasive electrophysiological testing were dissected out, embedded in paraffin, and sectioned in a paramodiolar plane at a thickness of 6 μm. Every 10th section was mounted on a slide (total of 10 slides). Immunohistochemical staining for calretinin or connexin 26 and 30 was then conducted. To this end, the sections were deparaffinized, washed with PBS, and then blocked in 1% bovine serum albumin (fraction V) and 1% normal goat serum in PBS plus 0.2% Triton X-100 (PBS/T). After rinsing with PBS/T, the sections were incubated with either rabbit anti-calretinin (1:500, EMD Millipore, Billerica, MA), rabbit anti-connexin 26 (1:200, Life Technologies, Carlsbad, CA), or mouse anti-connexin 30 (1:200, Life technologies, Carlsbad, CA) overnight. For calretinin immunolabeling, a biotinylated anti-rabbit IgG (1:200, Vector Laboratories, Inc. Burlingame, CA) was applied to the slides for 1 hour at room temperature, and Vectastain ABC and DAB kits (Vector Laboratories, Inc. Burlingame, CA) were then used for visualization. Immuno-positive cells had a dark brown reaction product. Methyl green was used for nuclear counter staining. For connexin 26 and 30 immunolabeling, the antibody-incubated sections were washed and immunolabeled with Alexa Fluor^®^ 488 donkey anti-mouse IgG and Alexa Fluor^®^ 594 donkey anti-rabbit IgG (1:1000, Life technologies, Co. Grand island, NY). DAPI was used to label nuclei. Fluorescence images were collected with an Olympus BX51 microscope (Melville, NY). Representative images were also collected with a Leica SP2 confocal microscope (Heidenberg, Germany). Six cochleae were analyzed for each treatment group and sampling interval, except for the 10d Normal and Treated groups and the 180d treated group. To assess the specificity of each immunolabeling, negative controls were prepared by omitting the primary antibody incubation step.

Measurement of the relative fluorescence intensity of connexin 26 and 30 signal in the organ of Corti and the spiral ligament was conducted with LAS AF Lite software (Leica Microsystems CMS GmbH, Heidenberg, Germany). Two to three images were collected from each turn from midmodiolar sections of each cochlea by fluorescence microscopy using the same camera settings. The images were taken only from midmodiolar sections so that a similar shape and size of the organ of Corti could be measured in all animals. The distance between two images was about 200–400 μm to ensure non-duplicate measurement. Relative fluorescence intensity was measured with the software by drawing a line along the border of either the organ of Corti or the spiral ligament [40].

A set of sections (adjacent sections used in the immunostaining) were stained by toluidine blue [41,42,43] to verify the number of the spiral ganglion cells. Expression of calretinin in the organ of Corti was examined via light microscopy (Olympus BX51, Melville, NY). Images of the spiral ganglion in the basal, second and third turns of each cochlea in 5–6 mid-modiolar sections were collected using the microscope. The number of cells, the size of the spiral ganglion, the number of nerve fibers, and the size of the spiral lamina were counted and measured using ImageJ software (National Institutes of Health). Fluorescence intensity and cell or nerve fiber densities from three turns were calculated (cells or fibers/mm^2^) and statistically analyzed (as detailed below).

### Group comparisons and statistical analysis

To facilitate group comparisons and statistical analysis, average threshold level and threshold shift were computed. According to the frequency response shape of ABR, the low frequency range (from 0.5 to 1 kHz) showed a less but similar threshold shift, while the frequency range from 2 to 8 kHz showed a similar threshold shift pattern. Therefore, the low frequency average was computed from 0.5 and 1 kHz, and the high frequency average was computed from 2, 4, 6, and 8 kHz for ABR, CAP and CM measurements. Similarly, the response pattern of DPOAE also showed a less dramatic shift at the low frequency range (from 1.1 to 1.6 kHz at F2) and a higher shift at the high frequency range (from 2.2 to 8.8 kHz at F2). Two-way ANOVAs, with one factor over different treatment groups, as well as another factor over different observation time groups, were used to assess overall differences in mean thresholds and threshold shifts, and amplitudes and amplitude shifts, that were measured by ABR, CAP, CM, and DPOAE analysis. Significant differences between groups were evaluated using Bonferroni post hoc tests. For the ABR and DPOAE threshold data from the180d post noise exposure group, the repeated measures one-way ANOVA was used to evaluate significant differences among in-group comparisons across the time interval. Significance levels were also analyzed using Bonferroni post hoc tests. Standard one-way ANOVA was used to test the overall difference between noise and treatment groups for the EP measurements. Significant differences in EP measurements between the different groups (including normal control) were tested using Bonferroni correction tests. In addition, correlations between the different outcome parameters were assessed using Pearson’s correlation coefficients. The number of ears included in the evaluations was displayed by n in each analysis.

## Results

### ABR and DPOAE analysis

**Fig 1:**
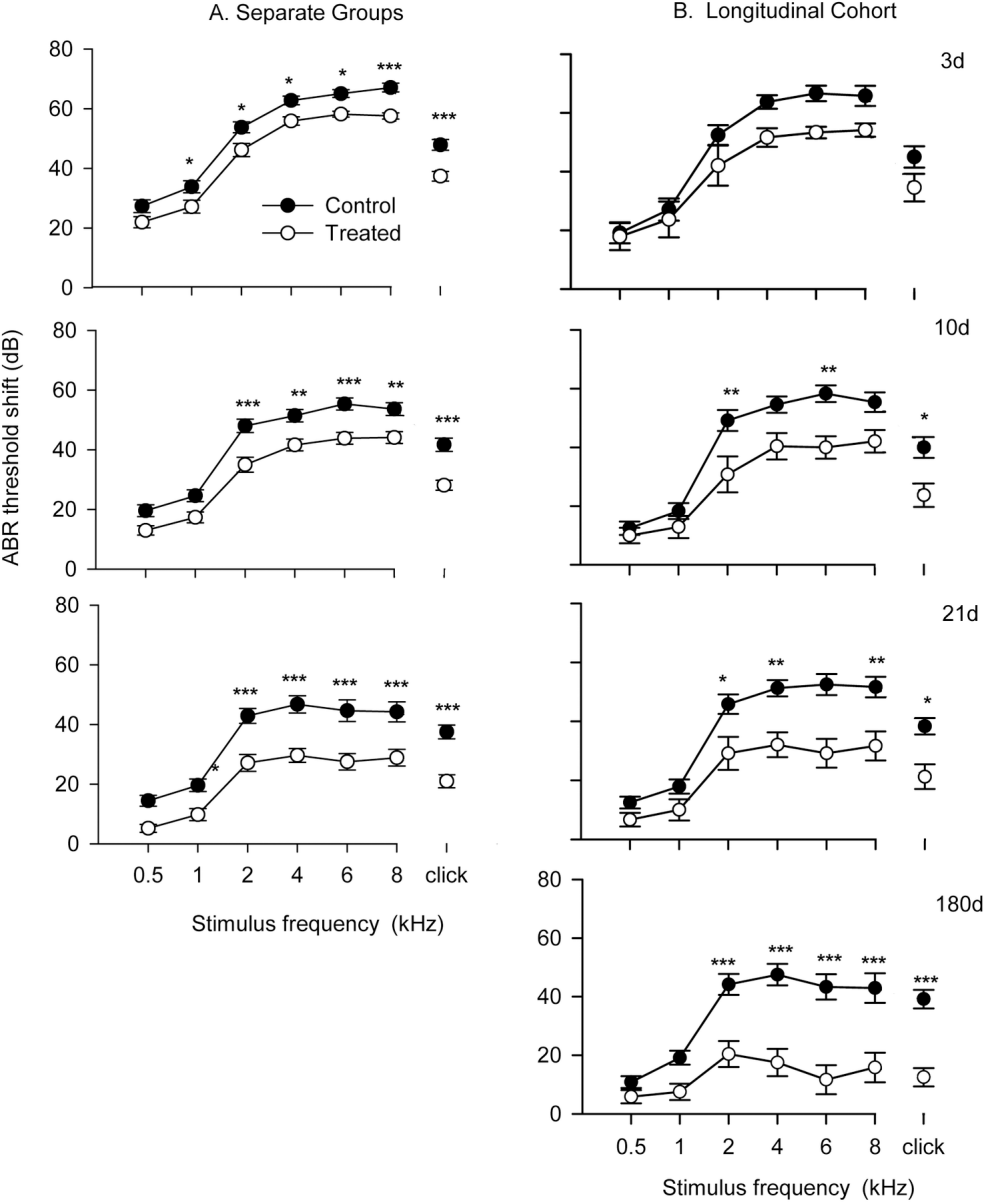
ABR threshold shift as a function of stimulus frequency for noise exposed-untreated (control) and—HPN-07 treated (treated) chinchilla 3, 10, 21 and 180 days after noise exposure. The number of chinchilla that were tested (both ears) at each time point (separate groups) were as follows: 3d (25); 10d (19); 21d (13); 180d (6). In the “longitudinal cohort study” the same six animals (12 ears) were tested at each time point. The symbols with horizontal bars represent the means and SEM of ABR threshold shift at individual stimulus frequency and Click (CLK) stimulus. The filled and unfilled symbols indicate noise exposed-untreated and HPN-07-treated groups respectively. Two-way ANOVA and Bonferroni Post Hoc test were applied (*, **, *** indicate significant level of P<0.05, 0.01 and 0.001 respectively). The data show that HPN-07 treatment reduced ABR threshold shifts to a similar degree at each frequency and time point in both experimental groups.

**Fig 2:**
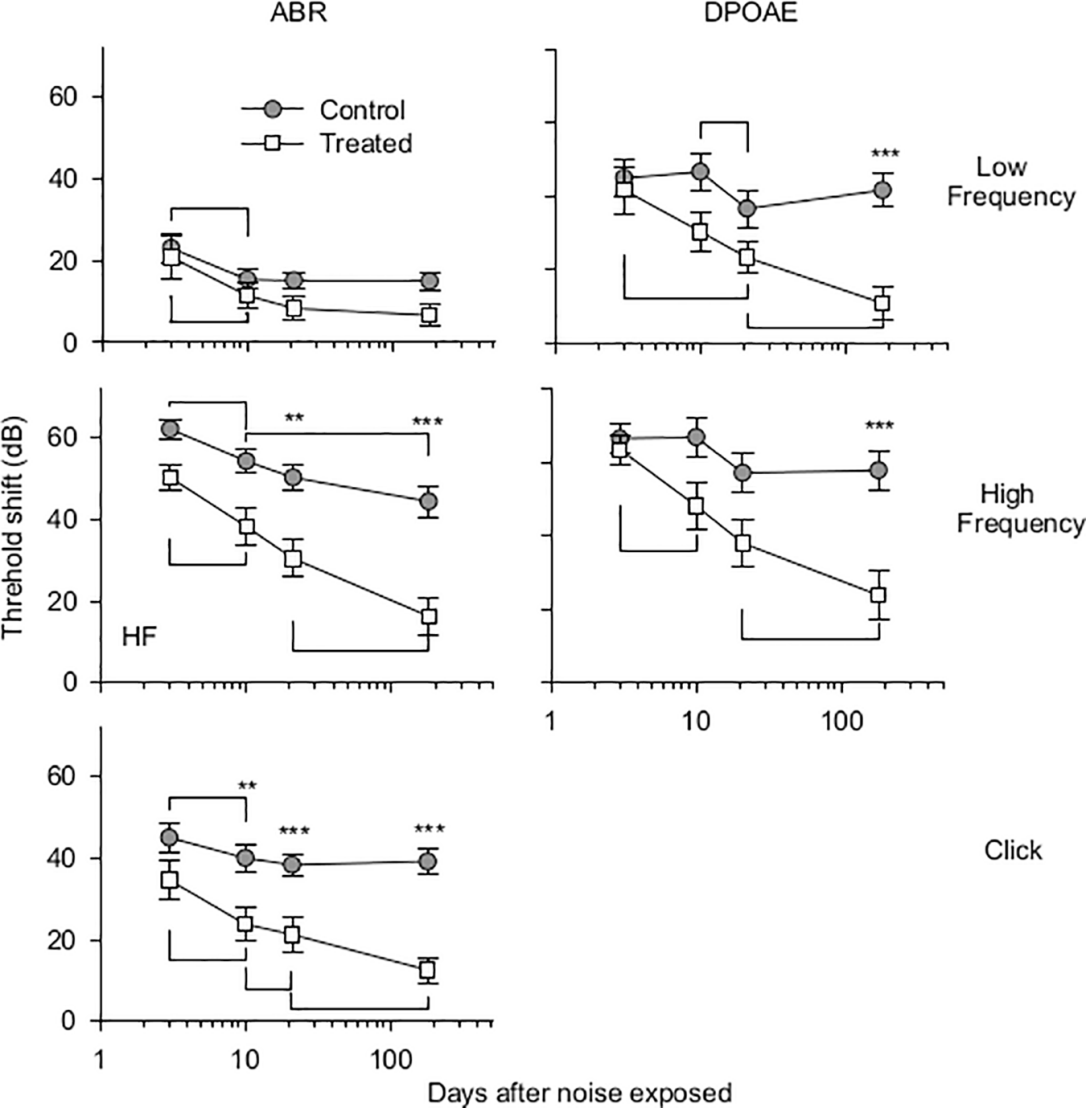
Time course of in-group ABR and DPOAE threshold shifts. The mean and SEM (vertical bars) for “click” (CLK), low frequency (LF), 0.5 and 2 kHz; and high frequency (HF), 2, 4, 6, 8 kHz ABR data were plotted for each time point. Two-way ANOVA and Bonferroni Post Hoc test were applied. The symbols of ** and*** indicate significant differences of *p* < 0.01and 0.001, respectively. Horizontal bars indicate significant differences of *p* < 0.05. Both the ABR and DPOAE threshold shifts of the HPN-07-treated group was significantly less than the untreated group at all recording intervals.

**Fig 3:**
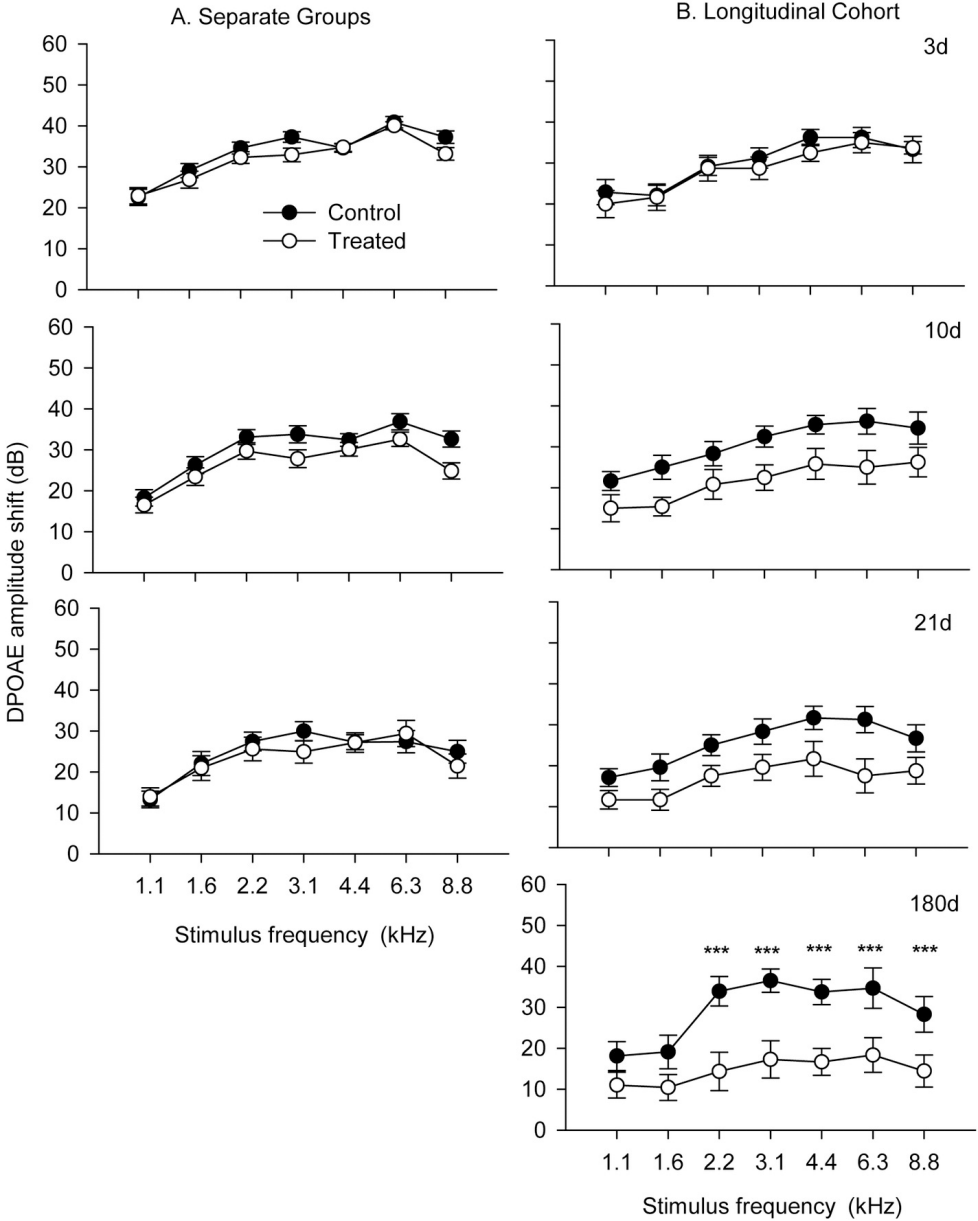
Distortion-product otoacoustic emission (DPOAE) amplitude shifts plotted in the same format as Fig 1. The number of ears at each time point is the same as ABR data. The symbols with vertical bars represent the means and SEM, respectively of DPOAE amplitude shifts at individual stimulus frequencies. Two-way ANOVA and Bonferroni Post Hoc test were applied. The symbols of *** indicate significance levels of *p* < 0.001.

The potential protective effects of HPN-07 intervention on the electrophysiology of the cochlea were measured in chinchilla exposed to a six-hour damaging noise of 4 kHz octave band noise at 105 dB SPL. Post-AAT, chinchillas were either left untreated (control group) or received a three-day regimen of HPN-07 treatment (treated group), after which objective measures of auditory function were made for both groups at predetermined intervals over the course of 180 days. ABR, and DPOAE, threshold shifts (Figs 1 and 2 respectively) and DPOAE amplitudes shifts (Fig 3) of both treated and control animals gradually recovered over time. However, the degree of threshold recovery in the HPN-07-treated group was significantly greater, both at early and late recording time points, than that observed in the control group (Fig 2). Similar results were obtained for both the ABR and DPOAE data that was obtained by combining data obtained at each time point and for data obtained from the longitudinal cohort (Figs 1 and 3) in which the same animals were followed for the entire 180d time period. These results demonstrate that serial testing of the same animals did not affect subsequent test results and underscore the reproducibility of the data with respect to the therapeutic response in HPN-07-treated animals and the timing of functional recovery (Figs 1 and 3). The two-way ANOVA revealed that the overall differences in ABR threshold shift between untreated animals and HPN-07-treated animals across the entire time-course were statistically significant at frequencies, at and above 2 kHz (*p* < 0.01–0.001) (Fig 1). These distinguishing HPN-07-associatd effects on functional recovery were noted at 10, 21, and 180d post-noise exposure (Fig 2). By comparison with the ABR threshold shifts DPOAE threshold shifts did not show the same degree of recovery in the untreated group but did show similar enhanced recovery in the HPN-07 treated group in response to therapeutic intervention with HPN-07 (Fig 2).

### CAP, CM, and EP analysis

**Fig 4:**
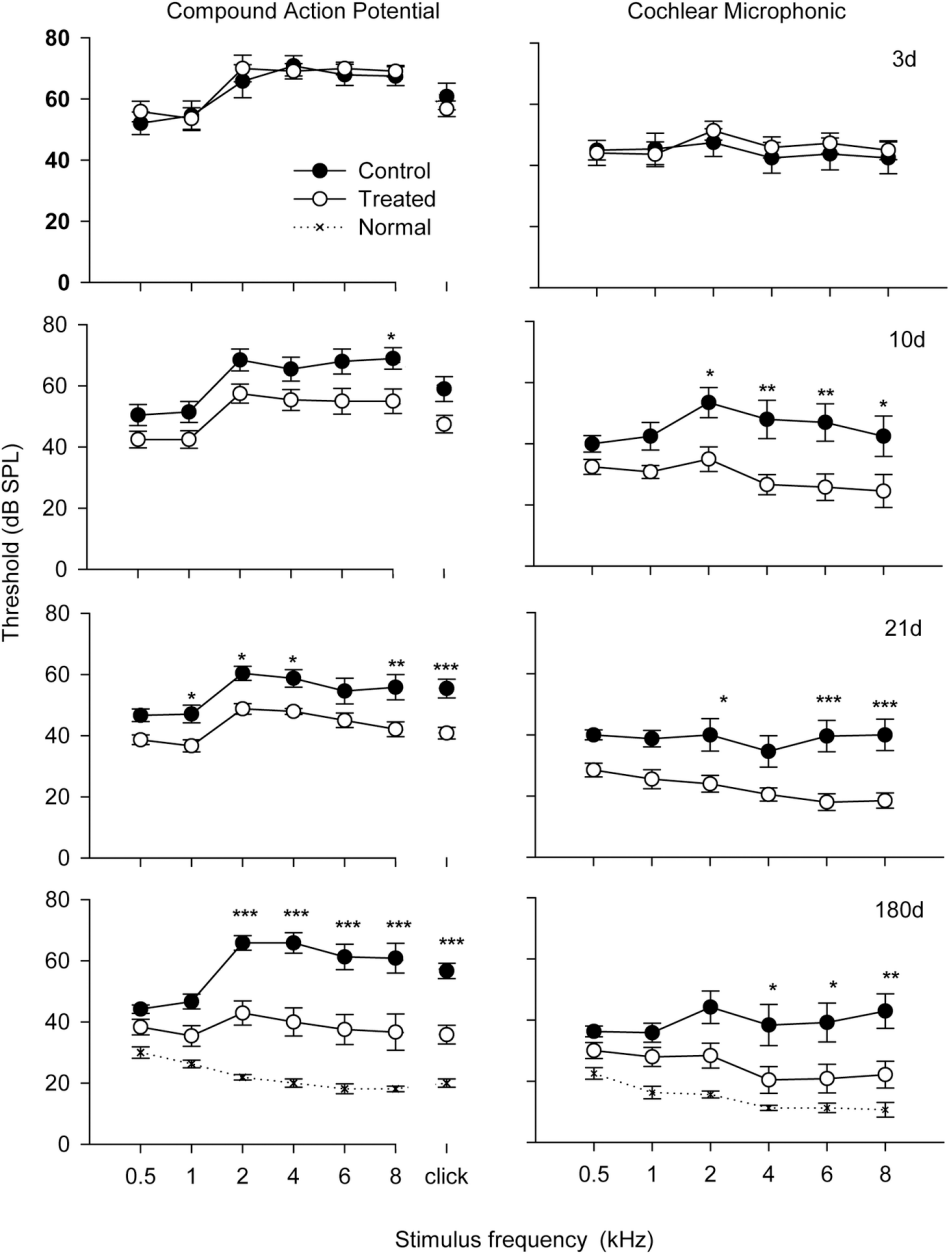
Compound action potential (CAP) and cochlear microphonic (CM) threshold change as a function of test frequency over different time groups plotted in the same format as Fig 1. Each sampling group contained readings from six animals. The results show a decrease in both CAP and CM thresholds for HPN-07 treated animals relative to control animals beginning at 10 days after blast exposure. Significant differences were detected by two-way ANOVA analysis. The symbols of *; **; *** indicate significance levels of *p* < 0.05, 0.01 and 0.001, respectively.

CAP and CM were performed as terminal procedures with an electrode placed at the base of the cochlea near the round window membrane. Both the CAP and CM (Fig 4) threshold values were elevated at 3d after noise exposure and remained at a similarly high level at each sampling point of the study thereafter in control, (untreated, noise-exposed) chinchilla. The CAP and CM threshold values for the HPN-07-treated group were indistinguishable from those of the control group at 3d post-injury. However, the HPN-07 treatment groups showed a progressive decrease in the thresholds of both of these electrophysiological metrics, beginning at 10d and approaching the threshold levels of the normal (naïve) group by 180d for all but the lowest two test frequencies.

**Fig 5:**
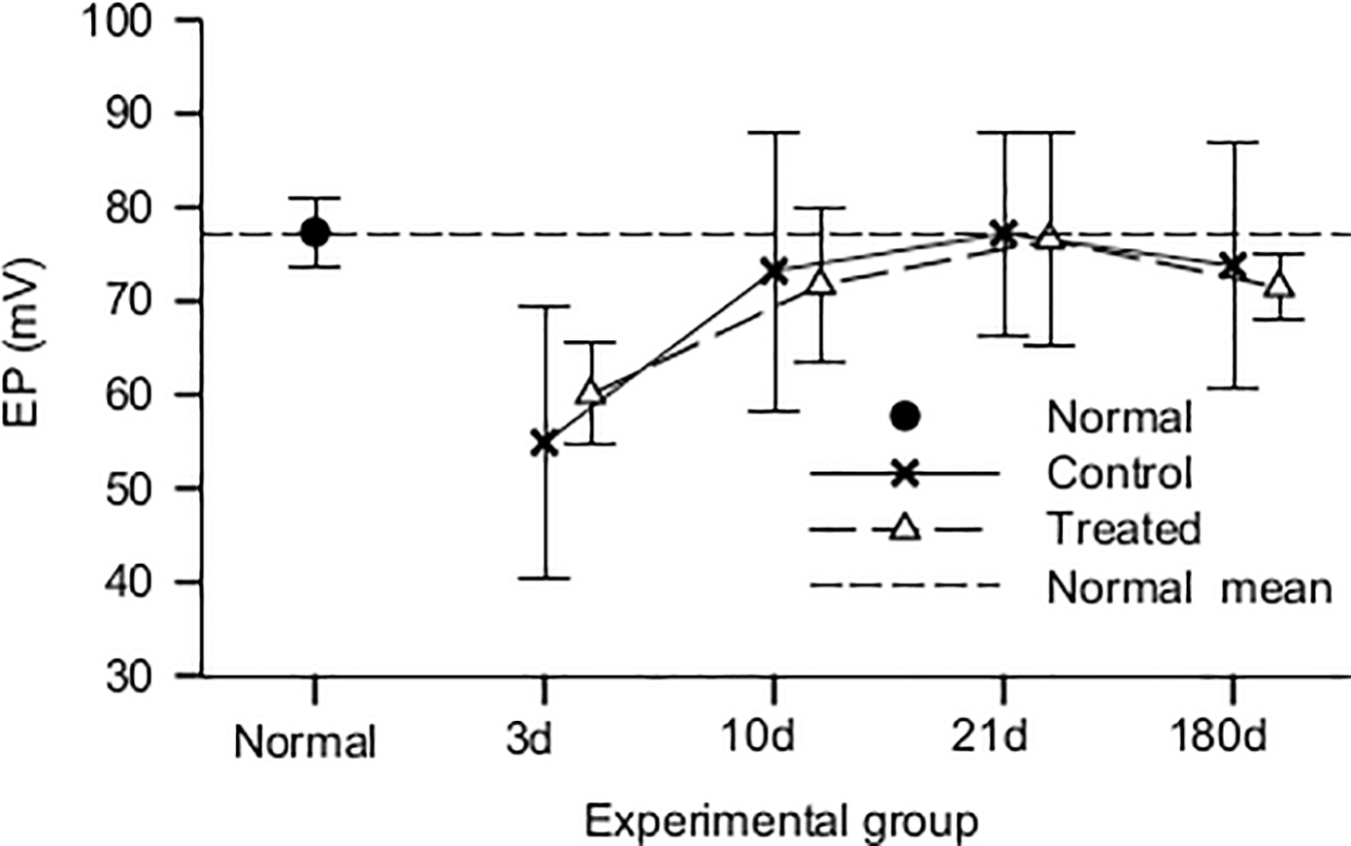
Mean endocochlear potential (EP) values for normal (naïve), (-control (noise only)), and treated (noise+HPN-07) groups (n = 6/group). The vertical bars indicate standard deviation of the data. EP values were significantly decreased in both the control and HPN-07-treated groups at 3d (days) after noise exposure compared to normal animals (*p* < 0.01) and recovered to the normal level 10d (days) after noise exposure (*p* > 0.05). The one-way ANOVA revealed that there were (is) no significant differences between the control and HPN-07-treated noise-exposed groups.

We observed that the mean EP values for both the control and HPN-07-treated groups at 3d post-noise exposure were lower than those of the normal (naïve) controls (Fig 5). The mean EP values for the normal animals were consistent with previously reported values of 75 mV measured in guinea pigs [3, 31,44]. However, the mean group averages of both control (untreated) and HPN-07-treated groups exhibited an EP recovery pattern that approached those of the normal animals beginning at 10d post-injury. The variation in EP scores, as represented by the standard deviation of values (n = 5), was markedly higher at 3d, 10d and 180d in the control group (14.6) than that measured within the HPN-07-treated group, which was the same as that of the normal controls (3.6).

**Fig 6:**
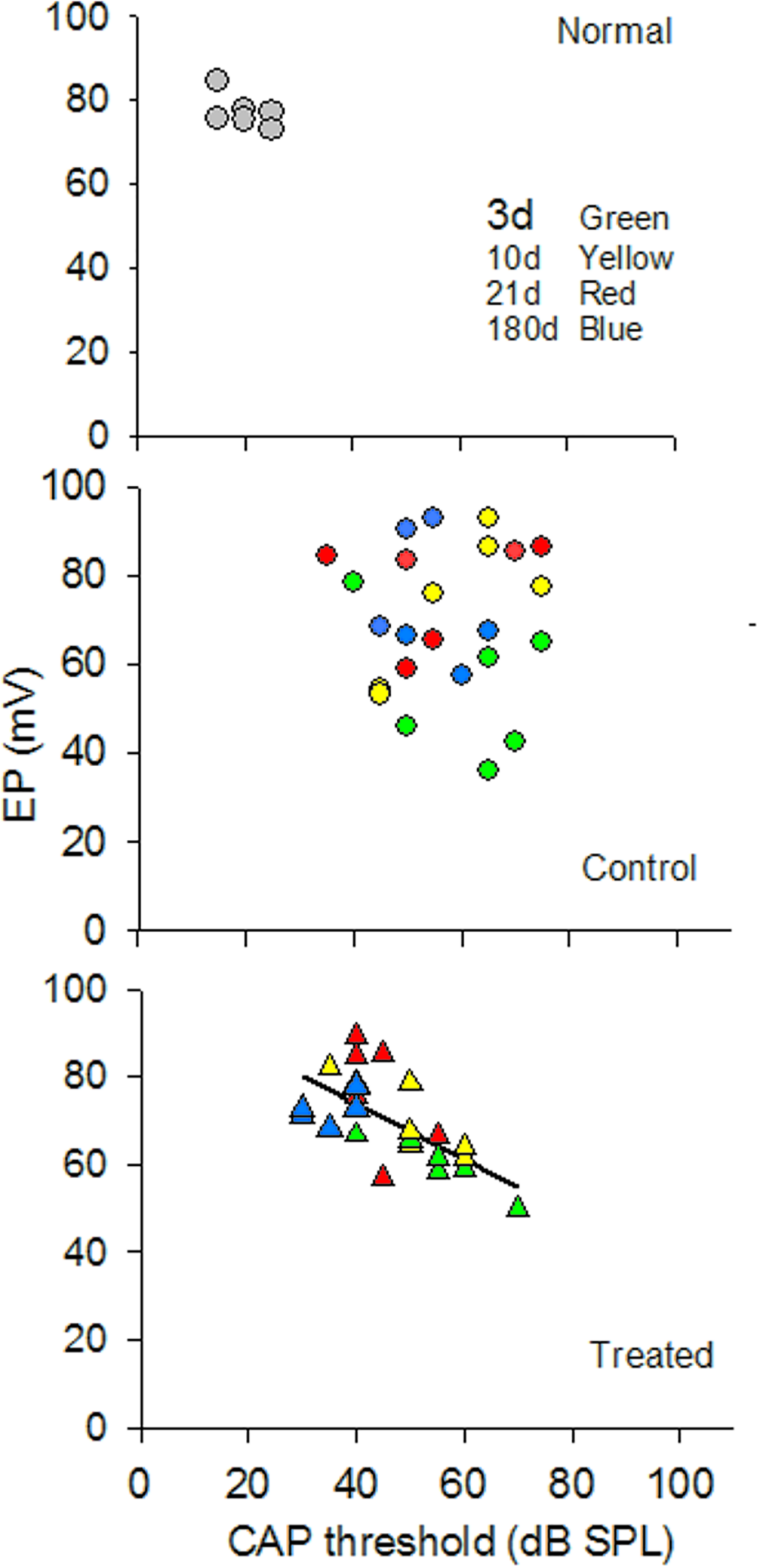
Bivariate analysis of the relationship between click-evoked CAP thresholds and EP for normal and noise-exposed chinchilla that were either treated with HPN-07 or untreated (control). Noise exposure resulted in a persistent disruption of the normal close correlation between EP and CAP values whereas in the HPN-07 treated animals a linear relationship (r = 0.656, n = 24, p = 0.005) was found after noise exposure between the two measurements with a gradual return to normal values.

**Fig 7:**
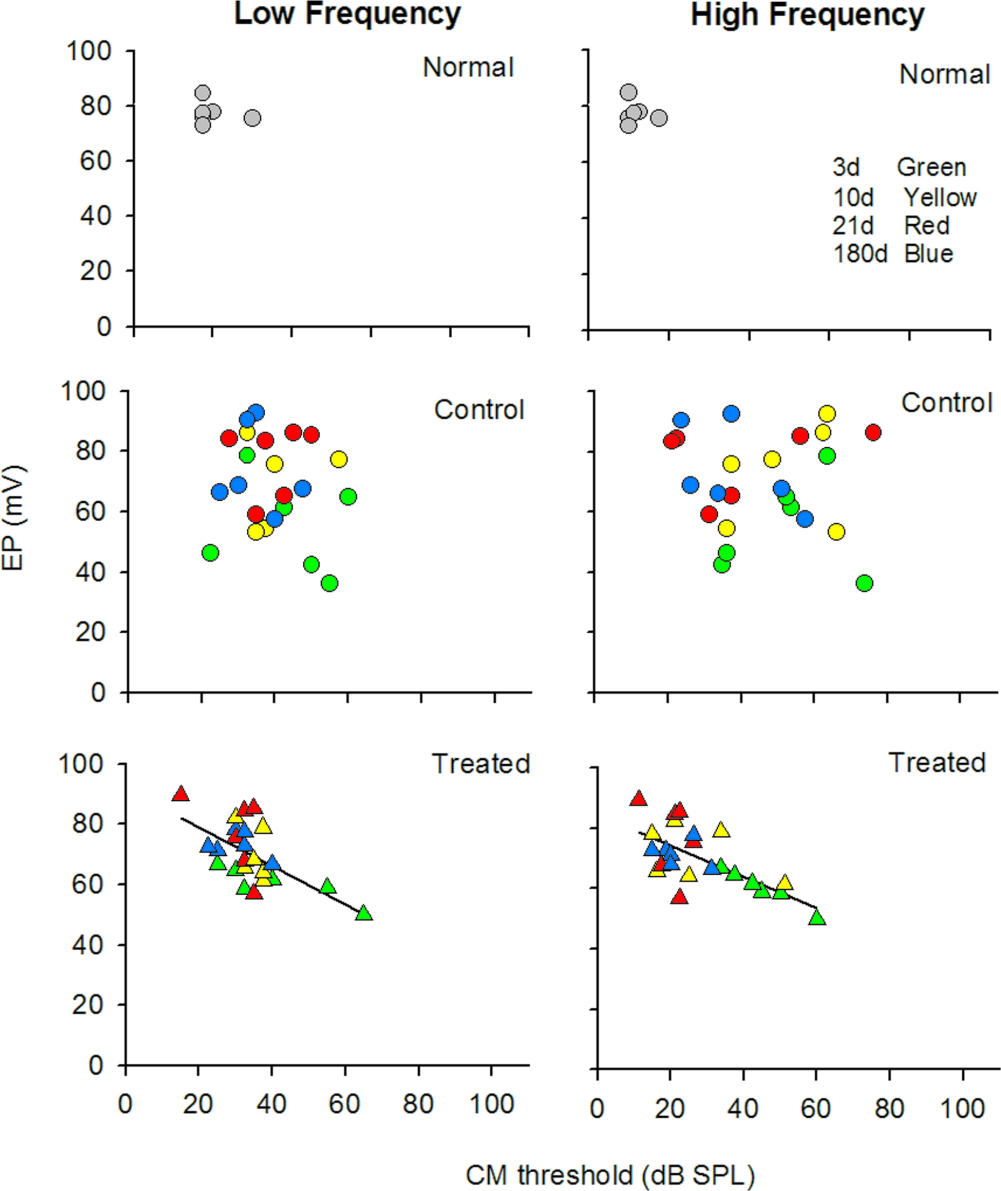
Bivariate analysis of the relationship between CM thresholds and EP for noise-exposed chinchilla in the HPN-07-treated and untreated groups (control). Data for low frequency (LF = 0.5 and 2 kHz); and high frequency (HF = 2, 4, 6, 8) kHz) were analyzed separately. Linear correlation analysis detected a correlation between EP and CM the in both the LF (r = 0.623, n = 24;) and HF (r = 0.676, n = 24) groups that were treated with HPN-07 following noise exposure but no correlation was detected in the untreated noise-exposed (n = 24) group.

The endocochlear potential (EP) is the positive voltage seen in the cochlear endolymphatic spaces which changes either positive or negative in response to sound and can affect the evoked potentials CM and CP. Because AAT can damage the cellular architecture that regulates the ion concentrations in the cochlea [44,45] we analyzed the electrophysiology data from individual animals to determine if there were correlations between EP and the two evoked potentials CAP and CM following AAT. Paired values of data from individual animals were plotted and correlations were assessed using Pearson’s correlation coefficients. The normal, animals showed tightgroupings of individual paired values for both the EP/CAP and EP/CM analyses (Figs 6 and 7). The AAT clearly disrupted the relationship between EP and CAP (Fig 6) and EP and CM (Fig 7) in the control animals, as evidenced by a seemingly random distribution of paired values and high inter-animal variability at each time point of the experiment. By contrast, in the HPN-07 treated animals the paired values conformed to a consistent linear correlation between EP and CAP values (Fig 6) and EP and CM (Fig 7). AAT appeared to alter the homeostatic relationship between EP and CAP or CM to variable degrees in untreated animals that persisted for 6 months. By contrast, a close relationship was maintained in HPN-07 treated animals, resulting in a coordinated return to normal homeostatic values.

### Histological findings

#### Cochlear hair cells

**Fig 8:**
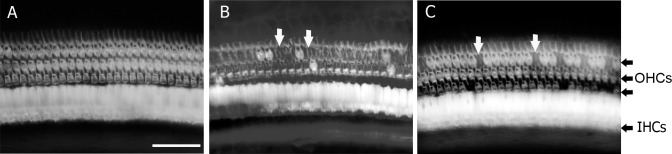
Representative photomicrographs of cochlear hair cells after phalloidin staining at about 70% of the distance from the apex in normal (naïve) (A), and control (untreated) (B) and treated chinchilla at 21 days after the blast exposure. Arrows show examples of missing outer hair cells (OHC) that have been replaced by scars. Scale bar = 50 μm.

**Fig 9:**
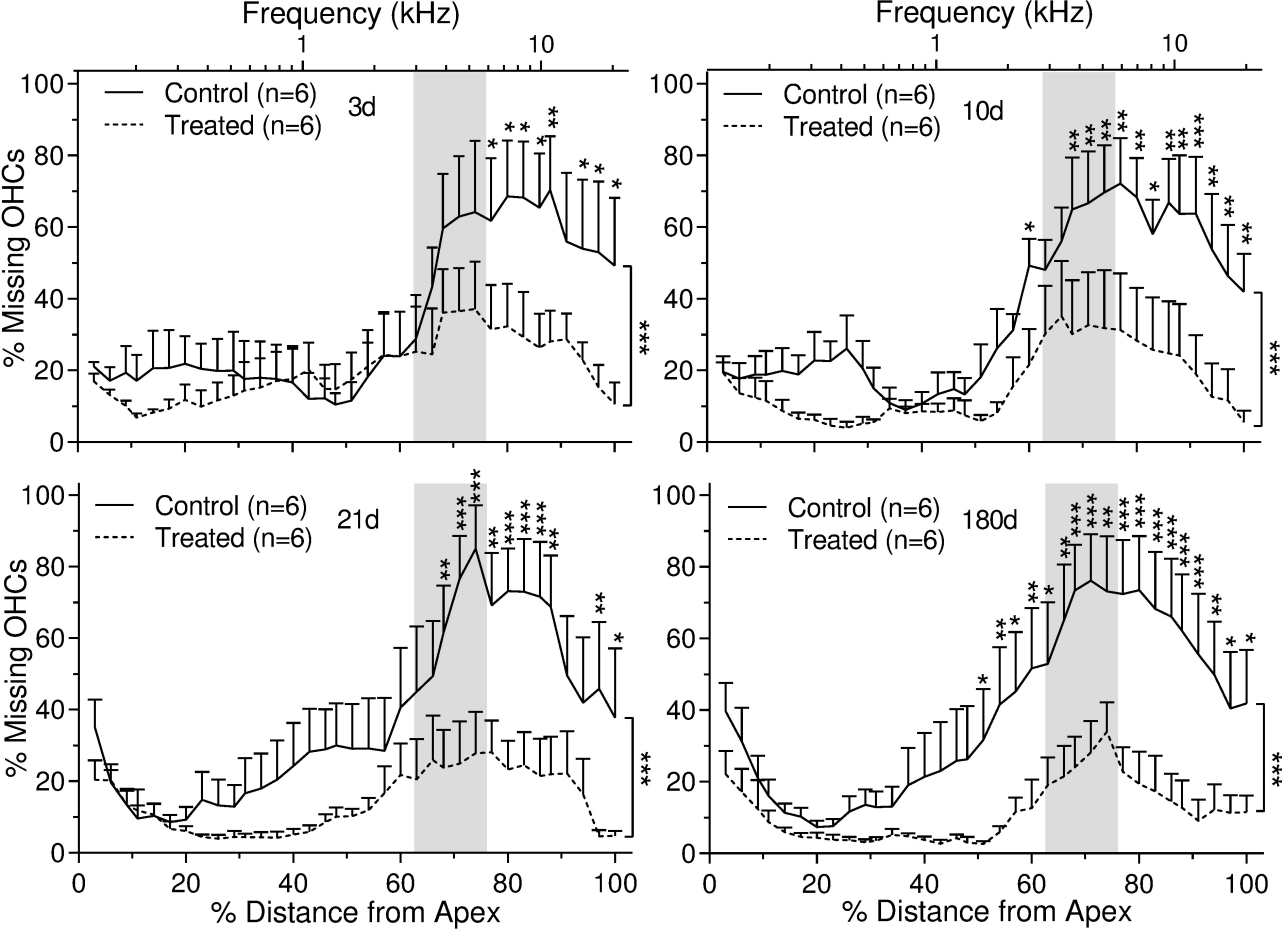
Percent of outer hair cell (OHC) loss as a function of percent distance (and frequency-specific tonotopic position) from the OC apex in untreated and HPN-07-treated chinchilla at 3 days (d), 10 d, 21 d, and 180 d after noise exposure. The data are plotted as mean ± SEM and “n” represents the number of cochleae. Vertical lines at right of figures show a significant difference of mean values for noise only "Control" and Treated" groups as determined by two- way ANOVA followed by Bonferroni post-tests: *, *p* < 0.05; **p < 0.01; ***p < 0.001. Shaded area demarcates the range of noise exposure centered at 4 kHz. Significantly less OHC loss was observed in the basal turn of the OC in the HPN-07 "Treated" group (*p* < 0.05, 0.01 or 0.001).

**Fig 10:**
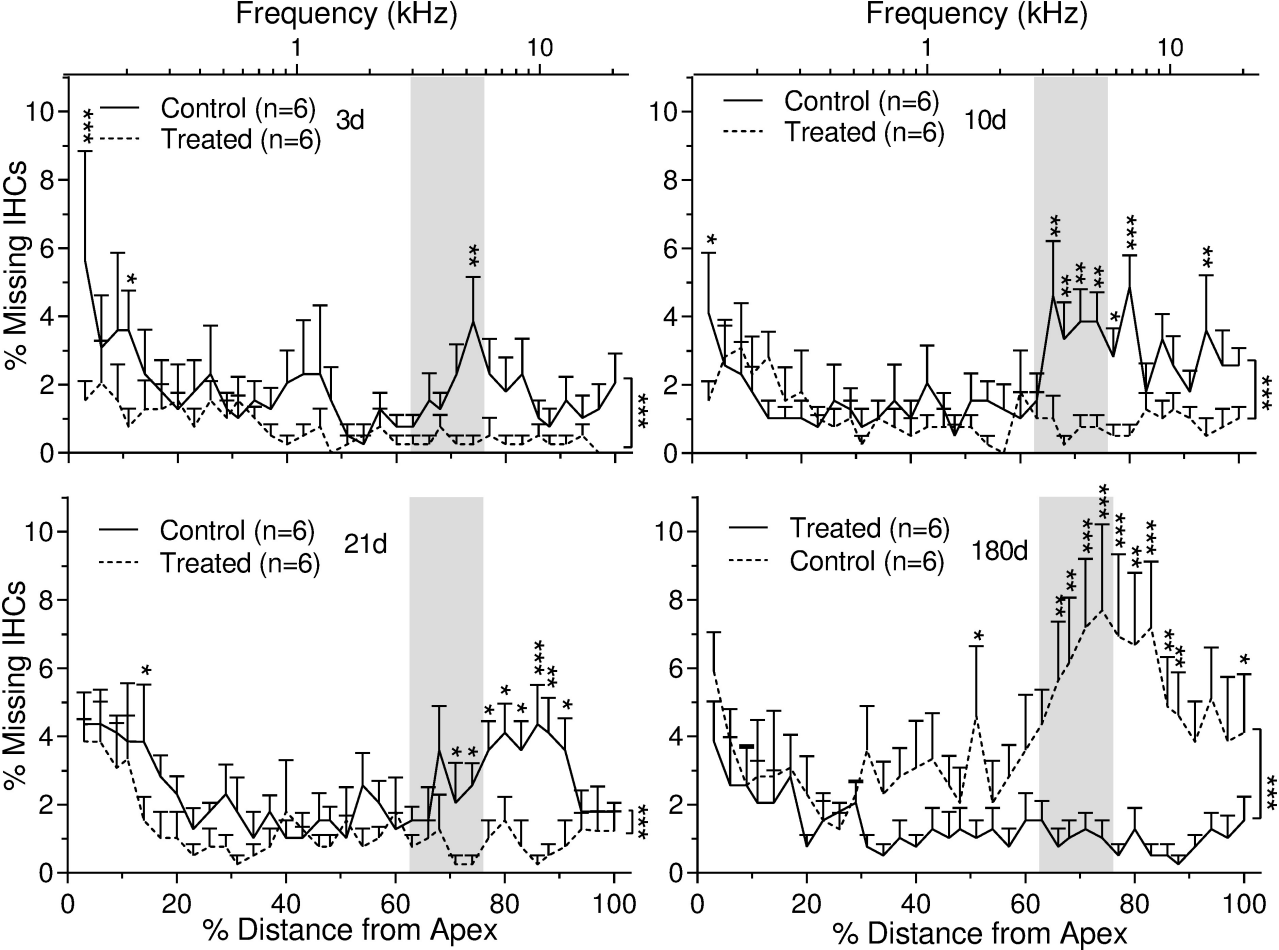
Percent of inner hair cell (IHC) loss as a function of percent distance (and tonotopic position in kHz) from the OC apex in untreated and HPN-07-treated chinchilla at 3 days, 10 days, 21 days and 6 months after noise exposure. Significantly less IHC loss was observed in the basal turn of the OC in the HPN-07 treatment group (*p* < 0.05, 0.01 or 0.001). The data are plotted as mean ± SEM, and “n”represents the number of cochleae. Vertical lines at right of figures show a significant difference of mean values for control (untreated) and HPN-07-treated" groups as determined by two-way ANOVA followed by Bonferroni post-tests: *p <0.05; **p < 0.01; ***p < 0.001. Shaded area demarcates the range of noise exposure centered at 4 kHz.

Surfaces preparations of cochlea were used to count both inner and outer hair cells across the entire basal membrane (Fig 8). The acoustic insult in this study produced pronounced and progressive outer (Fig 9) and inner (Fig 10) hair cell loss. This hair cell loss was most severe along the basal turns of the cochleae and appeared to extend apically with time. The HPN-07 treatment robustly reduced both OHC (Fig 9) and IHC losses (Fig 10) and seemingly prevented the ongoing injury observed to extend longitudinally in the control (untreated) animals. At 3d post-AAT, for instance, approximately 60% of the OHCs were missing in control animals within the basal turn and grading apically to the 4 kHz tonotopic region corresponding the frequency of insult noise, while HPN-07 treatment reduced the initial OHC loss by approximately 50% at this sampling interval (i.e. day 3) and, subsequently, blocked the apical migration of the lesion observed at later time points in untreated animals (Fig 9). Moreover, the extent of IHC loss in high frequency tonotopic positions progressively increased between 21d and 180d post-injury, while in contrast, the IHC loss in these same cochlear regions of theHPN-07 treated groups was virtually eliminated (Fig 10). Taken together, these results demonstrate that HPN-07 treatment provided a high degree of protection against both short- and long-term losses of both OHCs and IHCs in response to the AAT.

#### Calretinin-positive nerve fiber densities in the spiral lamina

**Fig 11:**
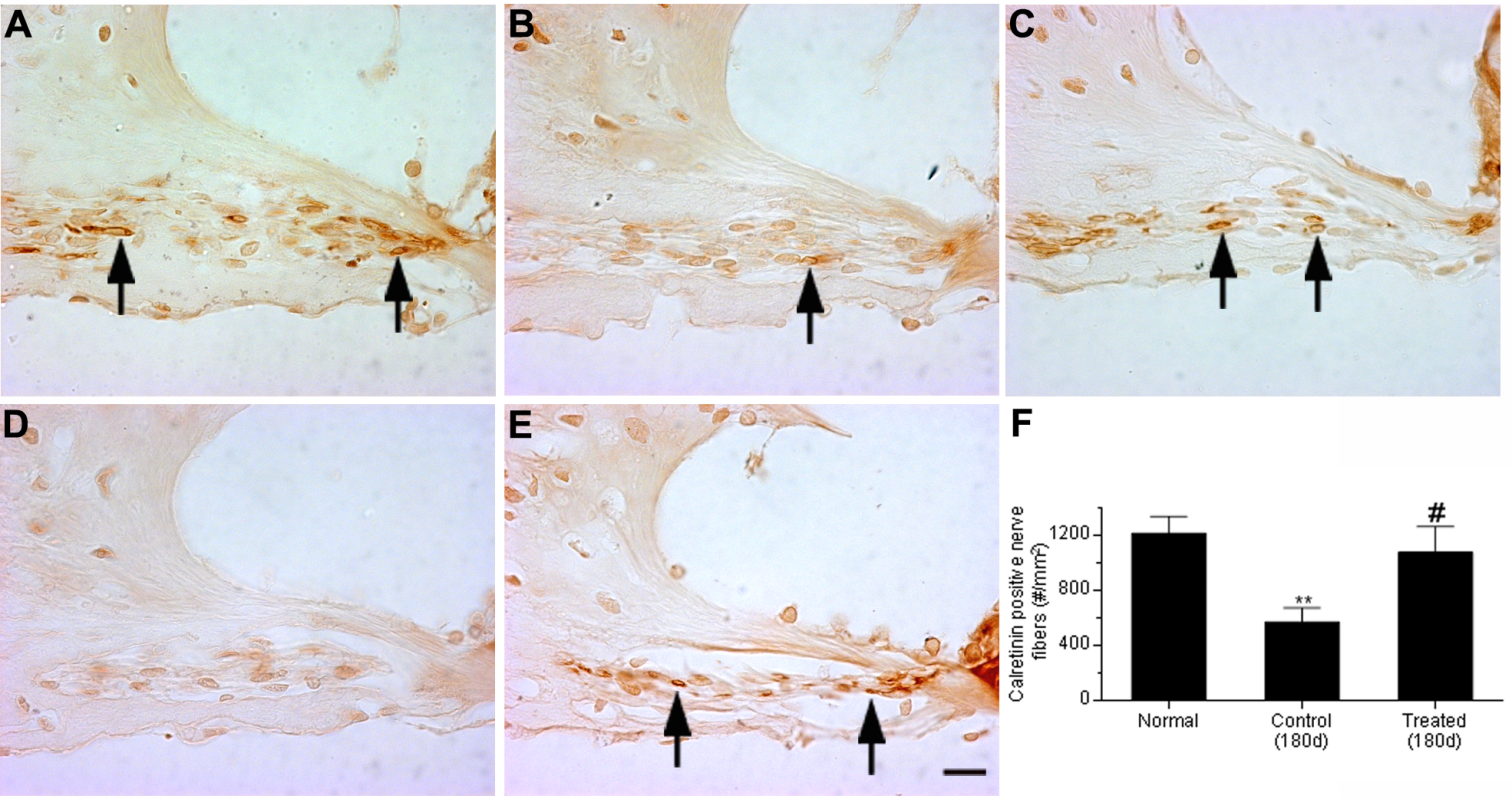
Calretinin staining in afferent nerve fibers in the middle turn spiral lamina of chinchilla. Representative images are shown from normal (naïve) animals (arrows in A), at 21 days after noise exposure in control (untreated animals) (B), at 21 days after noise exposure in HPN-07-treated animals (C), at 180 days after noise exposure in control animals (D) or at 180 days after noise exposure in HPN-07 treated animals (E). Fewer calretinin positive afferent nerve fibers were observed in the spiral lamina of chinchilla of noise exposure control groups (B and D). Density of calretinin positive afferent nerve fibers in the spiral lamina of chinchilla was measured in the middle and basal turns and statistically analyzed (F). Significantly decreased densities of calretinin-positive nerve fibers were measured in the spiral lamina at 6 months (**, *p* < 0.01) after noise exposure in the control group compared to the HPN-07 treated group, which was similar to that of the normal (naïve) group. (#, *p <* 0.0*5*). Scale bar = 10 μm in E for A-E.

Calretinin is a protein expressed by spiral ganglion neurons and is localized to the afferent nerve fibers that extend through the spiral lamina (SL) and terminate as nerve endings located under IHCs [46,47]. To evaluate the effects of the AAT on afferent nerve fiber density, calretinin-positive nerve fiber densities in the spiral lamina were quantified in the middle and basal turns of cochleae at 21d and 180d post-noise exposure and compared with that of cochleae from normal control ears. Compared to normal (naïve) animals (Fig 11A), fewer calretinin-positive nerve fibers were observed in the control (untreated, noise-exposed) group at both time points (Fig 11B and 11D). In contrast, the calretinin-positive nerve fiber densities observed in the cochleae from noise-exposed chinchillas that received HPN-07 treatment more closely mirrored those observed in normal animals (Fig 11C and 11E).

Quantitative analyses of these calretinin-positive afferent nerve fiber densities from normal animals and noise-exposed control or treated animals at the terminal, 180-day, time point after noise exposure are shown in Fig 11F. At 21 and 180 days post-noise exposure, the calretinin-positive nerve fiber densities in the SL were significantly reduced in noise-exposed animals compared to normal controls (*p* < 0.01), with only approximately 50% of the original observed density present. However, the calretinin-positive nerve fiber densities in noise-exposed animals that were treated with HPN-07 were significantly higher than those quantified in control (untreated, noise-exposed animals) (*p* < 0.05 and were similar to the calretinin stain densities quantified in normal animals, *p* > 0.05). These results indicate that early HPN-07 treatment prevented the progressive neuropathological response that ultimately culminated in the loss of calretinin-positive nerve fibers in the SL observed at 180 days after noise exposure in control animals.

#### Neurons in the spiral ganglion

NeuN is a nuclear biomarker for mature neurons in the spiral ganglia (SG) and was employed to histologically evaluate whether our AAT model induced neuron loss in the SG. No significant difference was observed in the number of Neu N-positive cells in the SG of normal (naïve) and noise exposed controls or treated chinchillas at the terminal sampling time point of 180d after the AAT (The neuron densities in the basal and middle turns were 177.27 ± 15.35, 177.73 ± 13.29 and 146.02 ± 10.1 mm^2^ for normal, control and treated groups, respectively, all *p* > 0.0). Furthermore, the total number of neurons in the spiral ganglion (toluidine blue positive), were found to be statistically identical in all three experiments groups (The neuron densities in the basal and middle turns were 273.45 ± 16.94, 275.59 ± 10.55 and 240.60 ± 7.25 mm^2^ for normal, control and treated groups, respectively, all *p* > 0.05). These results reveal that the neuronal cell death that is often purported to be associated with hair cell death had not progressed to a detectable threshold over the time course of our study.

#### Connexin expression in the cochlea

**Fig 12:**
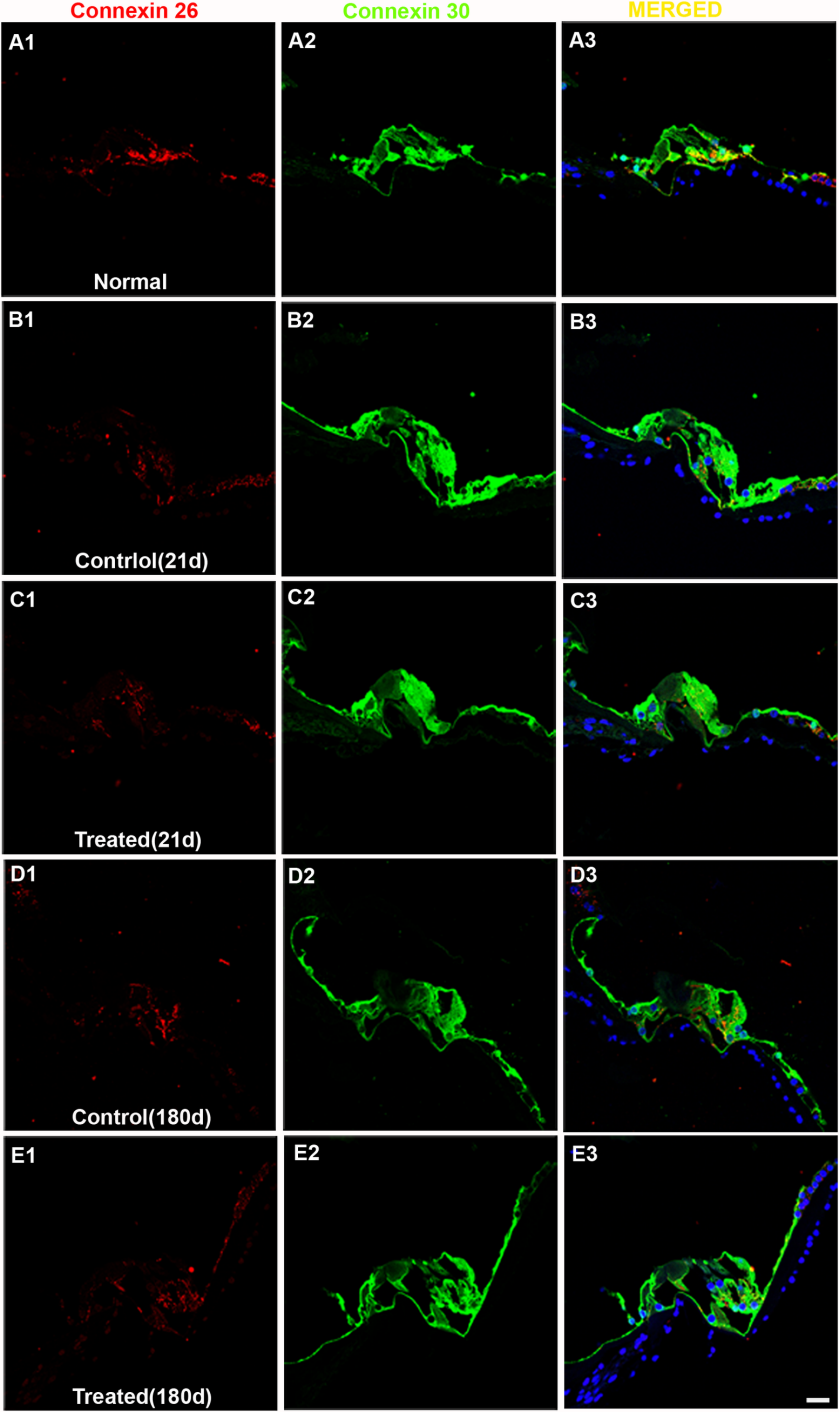
Representative confocal images of connexin 26 (Cx-26) and 30 (Cx-30) labeling in the OCs. Positive Cx-26 labeling (red) is observed in supporting cells in the OC of normal (naïve) animals (A1). Positive CX-30 labeling (green) is observed in all cells in the OC of normal animals (A2). Merged images are shown in column 3. Decreased expression of Cx-26 is observed in the OCs of control (noise exposed, untreated) or treated groups (B1-E1). The noise exposure did not change the expression of Cx-30 in the OCs all time points after noise exposure (B2-E2). Scale bar = 20 μm in E3 for A1-E3.

**Fig 13:**
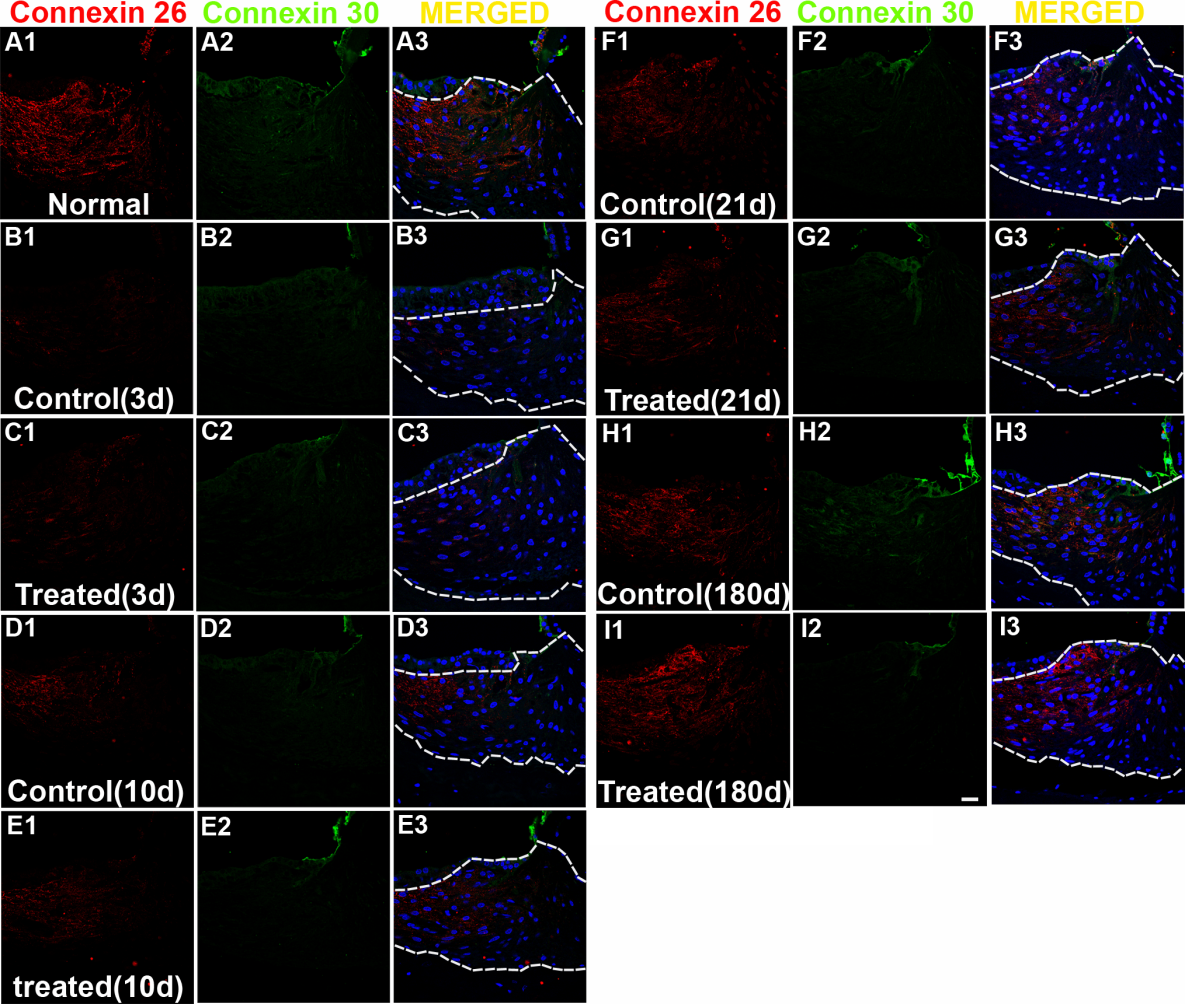
Representative confocal images of connexin 26 (red) and 30 (green) labeling collected from the spiral ligament. Nuclei of cells were stained by DAPI (blue). Merged images are shown in the column 3 (A3-I3, merged). Positive Connexin 26 labeling (red) is observed in fibrocytes in the spiral ligament of normal (naïve) controls (A1). Decreased connexin 26 labeling (column 1) is observed in the spiral ligament of chinchilla all time points after noise exposure (B-H) except in the treated group at 6 months after noise exposure(I). No or very low CX-30 labeling is observed in fibrocytes of the spiral ligament of chinchilla while the root cells show positive Cx-30 labeling (A2-I2). Scale bar = 20 μm in I3 for A1-I3.

Connexin 26 (Cx-26) is a major gap junction protein and plays a crucial role in the recirculation of K^+^ ions in the cochlea [48]. Consistent with previous studies [49,50] strong positive staining of connexin 26 was observed in fibrocytes in the spiral limbus, basal cells in the stria vascularis, type II and VI fibrocytes in the spiral ligament, and Deiters cells in the OCs of normal (naïve) animals (Figs 12 and 13). In contrast, relatively weak Cx-26-positive staining was seen in inner sulcus cells and interdental cells in the spiral limbus, outer sulcus cells in the spiral ligament, as well as Claudius cells in the OCs of normal chinchillas (Figs 12A1 and 13).

**Fig 14:**
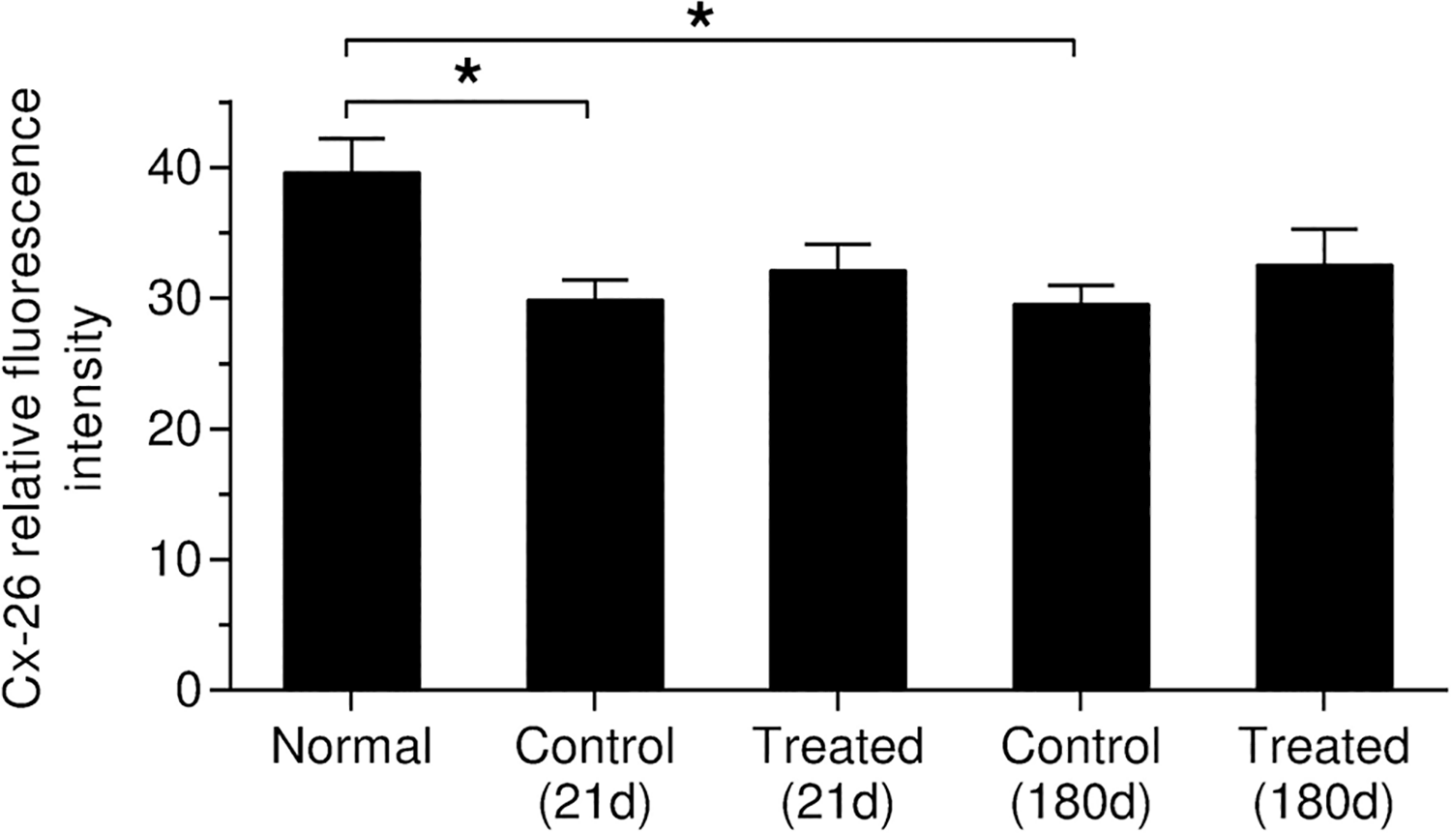
Connexin 26 relative fluorescence intensity in the OC was measured and statistically analyzed. Both noise exposed treated and control (untreated) groups showed a similar significant decrease in Cx-26 expression at 21 days and 6 months after noise exposure compared to the normal (naïve) animals (*p* < 0.05).

An apparent reduction of Cx-26 expression was observed in the OC following AAT in both control (untreated) and HPN-07-treated animals at 21 and 180d after noise exposure (Fig 12). No significant change in Cx-26 expression was detected in the OC at earlier sampling intervals (3 and 10d) after noise exposure (all *p* > 0.05). Formal signal intensity quantification confirmed a similar significant reduction of Cx-26 intensity in the OCs of both untreated (control) and HPN-07-treated animals at 21 and 180d after noise exposure (Fig 14, all *p* < 0.05). These data indicate that the AAT-induced reduction in Cx-26 levels were not mitigated by HPN-07 treatment and furthermore that the long-term effects of the AAT on gap junction integrity in OCs was not reliably ameliorated by the treatment.

In comparison to normal (naïve) animals (Fig 13A1), decreased Cx-26 labeling was also observed in the spiral ligament of chinchilla at 3d, 10d, and 21d after noise exposure in both control and HPN-07-treated animals (Fig 13B–13G). However, partial recovery was evident at 180d after noise exposure in both the control and HPN-07 treated groups (Figs 13 and 11).

**Fig 15:**
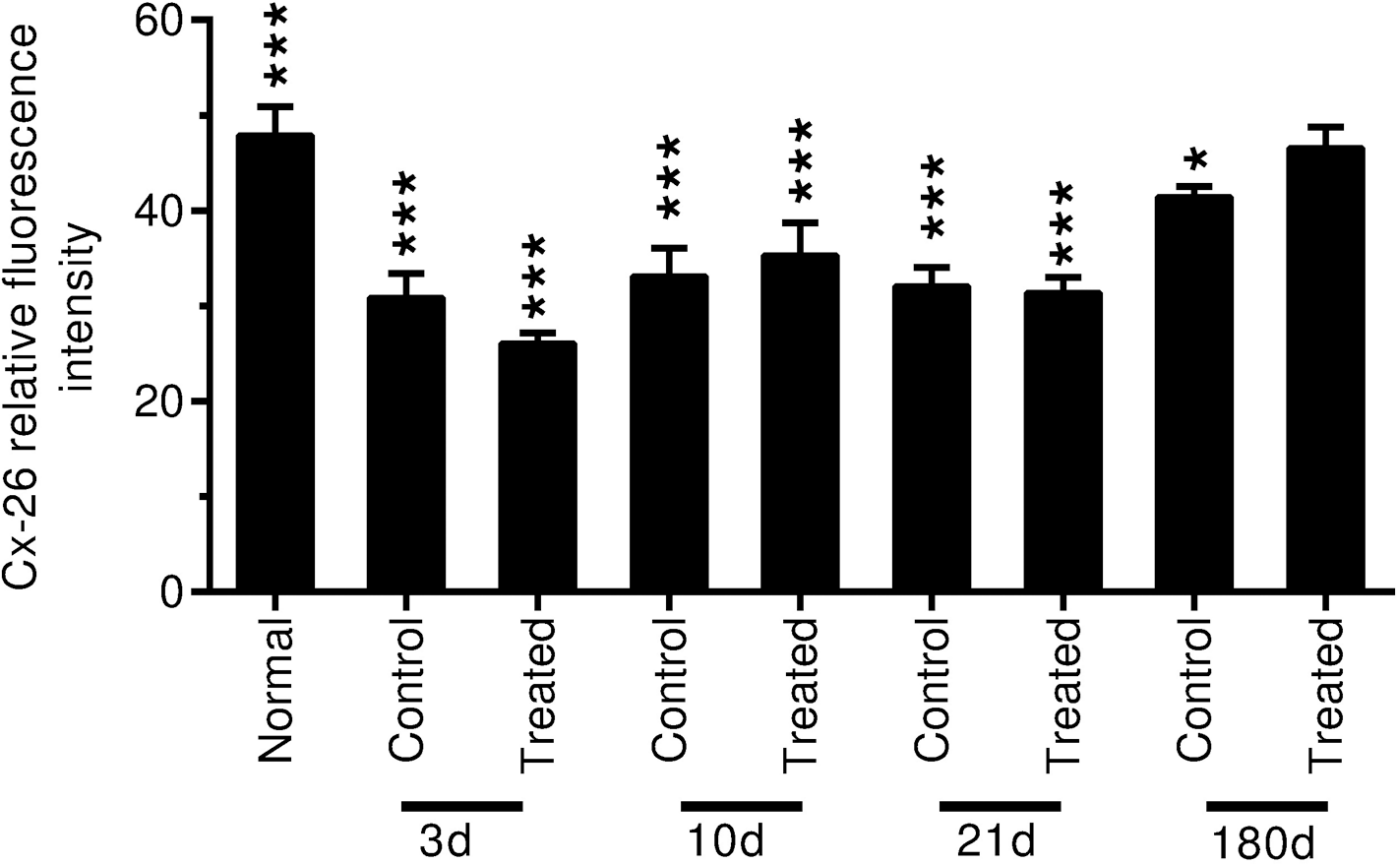
Connexin 26 relative fluorescence intensity in the spiral ligament in the middle and basal turns is measured and statistically analyzed. Significantly decreased expression of Cx-26 is observed in the spiral ligament of control and treated animals in the noise exposure groups from specimens collected at 3 to 21 days after noise exposure (***p < 0.001). Significant decreased Cx-26 expression is also observed in the control group 6 months after noise exposure (*p* < 0.05). No significant change is observed in Cx-26 expression in the spiral ligament of the treated group 6 months after noise exposure compared to the normal (naïve)animals (*p* > 0.05). No significant difference is observed between control and treated groups at each point after noise exposure (*p* > 0.05).

To confirm these observations, the relative fluorescence intensity of Cx-26 immunolabeling in the spiral ligament in the middle and basal turns was measured and statistically analyzed (Fig 15). Compared to normal animals, significant decreases in Cx-26 labeling intensity were measured in the spiral ligament of both the control and HPN-07-treated groups from 3 to 21 days after noise exposure (*p* < 0.01 or 0.001) and at 180d post noise exposure the Cx-26 labeling intensity in both groups approximated that of the normal animals (*p* > 0.05).

**Fig 16:**
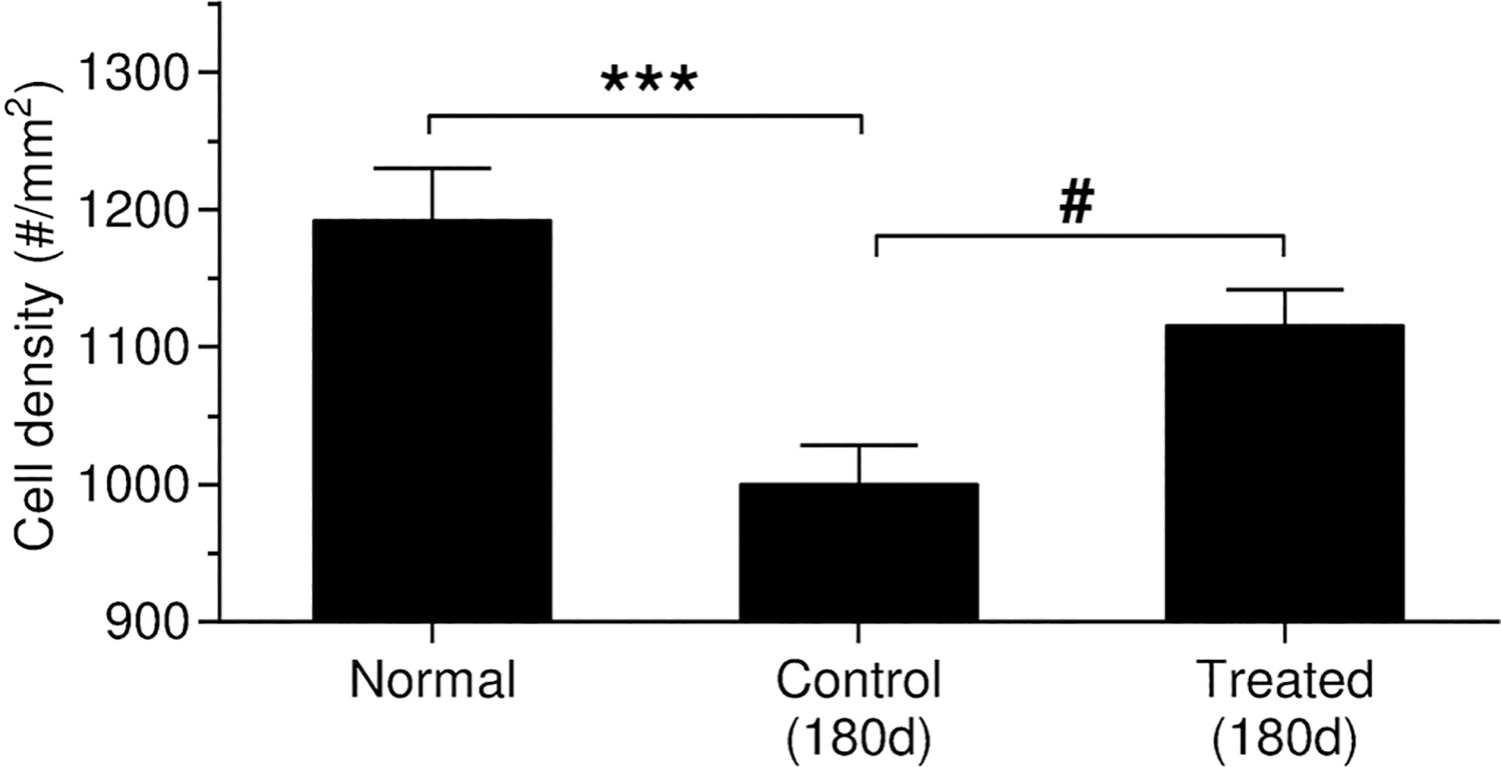
Fibrocyte density is calculated and statistically analyzed in the spiral ligament in the middle and basal turns. Significant lower cell density in the spiral ligament is only observed in the control (noise exposure, untreated group) 6 months after noise exposure (***, p < 0.001), and significant recovery is observed in the treated groups 6 months after noise exposure (#) compared to the control group (*p* < 0.05).

Diminished Cx-26 levels in the spiral ligament could be due to loss of fibrocytes after noise exposure [28]. Fibrocytes in the SL are also susceptible to oxidative stress after noise exposure, and antioxidant treatment can reduce this stress [40,51]. To address this possibility, the total number of cells stained by DAPI in the SL (DAPI positive nuclei within the dash lines in column 3 in Fig 13) were counted and statistically analyzed in the middle and basal turns (Fig 16). Significantly lower cell density in the spiral ligament was observed in the control group at 180d s after noise exposure (*p* < 0.001). However, any loss observed in the HPN-07-treated group was not significant compared to the control group (Fig 16, *p* < 0.05), suggesting that antioxidant treatment significantly reduced progressive fibrocyte loss in the spiral ligament after noise exposure. While reduced Cx-26 relative fluorescence intensity was observed in both treated and control groups at 3, 10, and 21d after noise exposure, fibrocyte loss was not observed at these time points (all *p* > 0.05). These results suggest that the reduced Cx-26 levels observed in the spiral ligament at these earlier sampling intervals were not due to loss of fibrocytes.

Another major gap junction protein in the cochlea is connexin 30 (Cx-30) [43]. The relative intensity of Cx-30 immunolabeling in the OC and spiral ligament was also evaluated and statistically analyzed (Figs 12 and 13). However, there was no apparent quantitative or qualitative differences (*p* > 0.05) in Cx-30 immunolabeling intensities between experimental groups in the OC (column 2 in Fig 12). Cx-30 labeling was either undetectable or very low in the spiral ligament of chinchilla under all conditions, making formal comparisons in this region difficult (column 2 in Fig 13). Collectively, these results indicate that, in contrast to Cx-26, the AAT model employed in this study did not significantly change the apparent expression levels of Cx-30 in the cochlea at any of the time points examined.

## Discussion

In this study, we demonstrated that therapeutic administration of, HPN-07, shortly after an AAT significantly protected against the progressive loss of cellular and electrophysiological integrity in the cochlea. The results compiled over a 180d recovery period allowed for a thorough examination of both short-term and long-term effects of the AAT and the degree of HPN-07-mediated protection of inner ear cytoarchitecture.

### Physiological changes in response to AAT

Following AAT, an initial recovery in ABR threshold shifts was observed by 10 d post-trauma in both the treated and untreated animals, however the extent of the measured recovery was significantly greater in the treated groups at the high frequencies and with click measurements. The elevated DPOAE thresholds, as indicated by threshold shifts, did not change between the 3d and 10d readings in the control group but were significantly reduced in the treated group at high frequencies. These results indicate that HPN-07 treatment reduced the temporary threshold shifts induced by acute noise exposure. Furthermore, the lack of improvement of the DPOAE measurements suggests that some of the ABR improvement in the controls was likely affected by recovery of neuronal function, which was enhanced by HPN-07 treatment [52]. Although we observed a small improvement in the control group in both ABR and DPOAE readings between 10 and 21 days post-AAT, the ABR improvement was significantly greater in the treated group. Surprisingly, between the 21d and 180 d readings, a significant further reduction of ABR and DPOAE threshold shifts was uniquely observed in the treated groups. The control animals showed no significant reduction of ABR or DPOAE over this same post-AAT interval. These findings were surprising because it is generally expected that threshold shifts will not improve beyond 10–21 days after AAT during which time the degree of hearing loss is considered permanent [53]. Given that the ABR threshold shifts in the treated groups were either less than or equal to the DPOAE threshold shifts over the same time intervals (Fig 3), it is probable that the measured HPN-07-mediated threshold improvement and longterm recovery is largely a function of the therapeutic effects of HPN-07 on the protection of OHCs from progressive loss following noise-induced injury [52].

The CAP, EP, and CM electrophysiological analyses provided additional information about the nature of the auditory defects induced by the AAT and the ameliorative effects of HPN-07 intervention. CM is the sound stimulus frequency following transducer potential that is primarily generated from OHCs, whereas CAP is the synchronized action potential of the auditory nerve [27,54]. Both CM and CAP are dependent upon a variety of histological and electrophysiological components, including the integrity of the cochlear partition, IHC and OHC function, magnitude of EP, and excitability of cochlear synapses [27,54]. Therefore, it would be expected that the two evoked potentials would follow the treatment effect on functional recovery as measured by ABR and DPOAE. However, although HPN-07 treated animals showed an initial reduction of the CAP and CM trauma-induced thresholds, very little recovery occurred after 10d post-trauma when a significant recovery of ABR and DPOAE threshold recovery was detected during this period. Taken together, these results suggest that the observed long-term functional recovery in the HPN-07-treated animals, as measured by the ABR and DPOAE, can only partially be accounted for by the mean values of the cochlear evoked potentials.

EP gives a measurement of the integrity of the cell membranes that form the barrier between the endolymph and the fluid in the organ of Corti [55]. We observed a decrease in the mean group EP values at 3d after noise exposure, with a return to the normal (naïve) group values between 10 and 21d post-injury in both the treated and control (untreated) groups. However, the inter-animal variability was greatly reduced in the treated group compared to the untreated group at 3, 10 and 180 days after AAT.

To further explore the effects of HPN-07, we performed bivariate analysis of the correlations between EP and CAP or CM responses because these measurements are dependent on multi-factorial electrophysiological components and architectural fidelity within the inner ear, as suggested by the reciprocal relationship between EP magnitude and CAP threshold in the mammalian inner ear [56]. Since such a correlation could be masked by averaging the data, we analyzed data from individual animals. We found a tight distribution of paired CM/EP and CAP/EP and values in control animals, consistent with the correlations between CAP and EP measurements in normal mice [32]. This close relationship between the different measurements was disrupted after noise exposure in the non-treated cohort and remained randomly dispersed for the duration of the 180d period of observation. In contrast, the paired values in the HPN-07-treated cohorts quickly returned to a tight distribution pattern.

The observed long-lasting idiosyncratic disruption of electrophysiological homeostasis in non-treated rats suggests that the clinical manifestations of noise exposure can be highly variable within a population receiving a similar level of noise exposure. A possible explanation of these findings may be found in previous studies, which have shown that perforations are produced in the reticular lamina in chinchilla even after only a modest noise exposure. [31,53]. An *in vivo* tracer analysis in chinchillas demonstrated that an intense noise exposure (4-kHz OBN, 108-dB SPL, 1.75 h) was sufficient to induce the formation of acute lesions within the reticular lamina, which led to a similar spatiotemporal EP recovery profile to that observed in our analyses [31]. Also, noise trauma-induced damage to the tight junctions in the lamina may exacerbate this altered electric potential profile, as noted below. Taken together, these results indicate that treatment with HPN-07 may act to preserve the structural integrity of the cochlea necessary for maintaining electric potentials, and the maintenance of more homeostatic correlation between EP and both CM and CAP [57].

### Cellular/molecular changes

In general, most of the OHC loss that we observed in untreated chinchilla at 3d post-trauma occurred at and above the tonotopic regions corresponding to the insult frequency. Progressive migration of OHC attrition to lower frequency regions was evident between 10 and 180d post-insult. A similar pattern of progressive OHC loss beyond the tonotopic region of injury has previously been documented in noise-exposed chinchilla [55]. Remarkably, we observed that OHC loss was not only significantly reduced in HPN-07 treated animals in tonotopic regions corresponding to the initial insult, but its subsequent apical migration was also blocked in the treated animals.

In contrast to OHC loss, which plateaued at about 10d post-insult, IHC loss continued longitudinally, with most of the loss occurring between 21 and 180d in the untreated animals. In contrast, less than 1% IHC loss was observed at all tonotopic positions in HPN-07-treated animals during the 180d study period. The progressive IHC loss observed in untreated, noise-exposed chinchilla may be attributable to ongoing inflammation or oxidative stress post-injury, both of which have been shown to be attenuated by HPN-07 treatment in other tissue injuries [14,48,58,59]. The observed protection of afferent nerve fibers in the treatment group may, thus, be an indirect effect of HC protection or a direct antioxidant effect on the nerve fibers themselves.

Our results revealed an approximate 50% decline in calretinin-positive neurites in control (untreated, noise-exposed) animals over the 180d study period whereas less than 10% neurite loss was observed over this same interval in HPN-07-treated animals. Preservation of calretinin-positive neurites in HPN-07-treated cochleae is consistent with the observed protective effects of HPN-07 on CAP, which measures neuronal output. However, CAP can also be affected by HC function, which was also preserved by HPN-07. Therefore, it is not possible to differentiate between the indirect effect of the antioxidant treatment on neurite protection mediated through HC survival versus direct effect on the nerve fibers themselves.

Previous immunohistochemical evaluations of Cx-26 and ultrastructural work in rat cochleae suggested that gap junctions serve as the structural basis for recycling endolymphatic potassium ions that pass through the sensory cells during the transduction process to maintain high K^+^ concentration (i.e. EP) [49,60]. However, disruption of cochlear gap junctions after an AAT has not been widely examined, even though it has been generally suspected [3,61]. Indeed, to our knowledge, only one such study has been reported to date [62]. In that study, Hsu and colleagues observed increased expression of Cx-26 in the lateral wall of the rat cochlea immediately after acute noise exposure. Consequently, it was surprising to find that although group mean values for EP recovered to pre-trauma magnitudes at 10d after injury, independent of treatment, the levels of Cx-26 in the OC and spiral ligament decreased in both cohorts beginning at 3d post-injury and persisting for up to 180d thereafter. However, the data for individual animals for EP-CAP/CM ratios (Figs 11 & 12) suggest that there remains a failure to return to the pre-acoustic trauma homeostatic electrical potential relationships even after 180ds which could be explained in part by Cx-26 loss suggestive of a disruption of the gap junctions.

Significant loss of fibrocytes was not observed in the spiral ligament after 21d following noise exposure. In a previous report, loss of type II fibrocytes was observed in murine cochlea two weeks after a 116 dB noise exposure [28]. This difference in timing of fibrocyte loss could be attributed to species differences or experimental conditions. In the present study, the reduced Cx-26 expression in fibrocytes among noise-exposed animals may account for some of the electrochemical abnormalities as measured by CM and CP. These fibrocytes have been postulated to play a critical role in potassium ion homeostasis [63]. Nonetheless, HPN-07 treatment significantly reduced fibrocyte loss in the spiral ligament, consistent with previous work from our lab and others in which antioxidants have been shown to protect against noise-induced attrition among this OC cell population [40,52].

Although the exact mechanisms of HPN-07's otoprotection is not known, it most likely functions as a free radical scavenger that suppresses the damaging effects of AAT-induced oxidative stress. Previous studies in our lab have established correlations with noise exposure and markers of oxidative stress and the ability of antioxidant treatment to reduce these effects. Chinchillas exposed to noise had elevated levels of 4-hydroxy-2-nonenal (4-HNE) in the cochlea and nitrotyrosine (NT) in the spiral ligament after noise exposure. Treatment shortly after noise exposure with the antioxidant drugs NAC and 4-OHPBN, a nitrone-based free radical trap similar to HPN-07, reduced the increase in levels of NT [35, 64]. Recently we have shown that treatment with HPN-07 and NAC after blast exposure significantly reduced the expression of a biomarker for oxidative DNA damage, 8-hydroxy-2’-deoxyguanosine (8-OHDG), in the spiral ganglion neurons of rats. [64]. Taken together, these findings along with those from other labs [5,6,8,9] give strong support for oxidative stress as a primary agent of cellular damage in the cochlea related to hearing loss. It should not be surprising, therefore, that HPN-07, with a phenyl ring that reacts with hydroxyl radicals, can have a significant effect on preventing such damage.

Previous studies in our lab have shown that AAT initiates ototoxic mechanisms that can be blocked by therapeutic interventions within a narrow window of time after noise exposure [33]. Surprisingly, the observation that HPN-07 treatment initiated within four hours after noise exposure and continued for 2 days has long-lasting effects, some of which only become evident 21–180 days later, is not readily explained from our evaluations but could be interpreted to suggest that HPN-07 may have the capacity to initiate repair mechanisms or a regenerative response among one or more components of the cellular architecture of the cochlea.

In summary, the physiological and histological findings presented here indicate that acute post-trauma intervention with HPN-07 as a single therapeutic agent promotes both acute and chronic recovery of hearing function. The data indicates that this functional recovery is related to the stabilization of the noise-induced histological and electrophysiological changes in the inner ear, especially the function of the HC. Overall, these results underscore that capacity of HPN-07 t to limit AAT-induced oxidative stress within the cellular framework of the auditory system and limit the induction of other pathological processes (e.g. inflammation) that have the capacity to perpetuate damage to the cytoarchitecture of the cochlea that have negative functional consequences.
